# “*Candidatus* Thermonerobacter thiotrophicus,” A Non-phototrophic Member of the *Bacteroidetes/Chlorobi* With Dissimilatory Sulfur Metabolism in Hot Spring Mat Communities

**DOI:** 10.3389/fmicb.2018.03159

**Published:** 2019-01-09

**Authors:** Vera Thiel, Amaya M. Garcia Costas, Nathaniel W. Fortney, Joval N. Martinez, Marcus Tank, Eric E. Roden, Eric S. Boyd, David M. Ward, Satoshi Hanada, Donald A. Bryant

**Affiliations:** ^1^Department of Biological Sciences, Tokyo Metropolitan University, Hachioji, Tokyo, Japan; ^2^Department of Biochemistry and Molecular Biology, The Pennsylvania State University, University Park, PA, United States; ^3^Department of Biology, Colorado State University–Pueblo, Pueblo, CO, United States; ^4^Department of Geoscience, University of Wisconsin–Madison, Madison, WI, United States; ^5^Department of Natural Sciences, University of St. La Salle, Bacolod, Philippines; ^6^Department of Microbiology and Immunology, Montana State University, Bozeman, MT, United States; ^7^Department of Land Resources and Environmental Sciences, Montana State University, Bozeman, MT, United States; ^8^Department of Chemistry and Biochemistry, Montana State University, Bozeman, MT, United States

**Keywords:** hot spring, microbial mat, sulfate reducing bacteria, *Bacteroidetes*/*Chlorobi*, metatranscriptome, metagenome, *dsrAB*

## Abstract

In this study we present evidence for a novel, thermophilic bacterium with dissimilatory sulfur metabolism, tentatively named “*Candidatus* Thermonerobacter thiotrophicus,” which is affiliated with the *Bacteroides/Ignavibacteria/Chlorobi* and which we predict to be a sulfate reducer. Dissimilatory sulfate reduction (DSR) is an important and ancient metabolic process for energy conservation with global importance for geochemical sulfur and carbon cycling. Characterized sulfate-reducing microorganisms (SRM) are found in a limited number of bacterial and archaeal phyla. However, based on highly diverse environmental *dsrAB* sequences, a variety of uncultivated and unidentified SRM must exist. The recent development of high-throughput sequencing methods allows the phylogenetic identification of some of these uncultured SRM. In this study, we identified a novel putative SRM inhabiting hot spring microbial mats that is a member of the OPB56 clade (“*Ca*. Kapabacteria”) within the *Bacteroidetes/Chlorobi* superphylum. Partial genomes for this new organism were retrieved from metagenomes from three different hot springs in Yellowstone National Park, United States, and Japan. Supporting the prediction of a sulfate-reducing metabolism for this organism during period of anoxia, diel metatranscriptomic analyses indicate highest relative transcript levels *in situ* for all DSR-related genes at night. The presence of terminal oxidases, which are transcribed during the day, further suggests that these organisms might also perform aerobic respiration. The relative phylogenetic proximity to the sulfur-oxidizing, chlorophototrophic *Chlorobi* further raises new questions about the evolution of dissimilatory sulfur metabolism.

## Introduction

Microbial sulfate respiration is an ancient metabolic process for energy conservation, which may have originated as early as 3.47 billion years ago (Shen et al., 2001; Wacey et al., 2011). Sulfate-reducing microorganisms (SRM) have global importance for biogeochemical sulfur and carbon cycling and are prevalent in both marine sediment (Bowles et al., 2014) and freshwater wetland ecosystems (Pester et al., 2012). It has been estimated that more than 50% of the organic carbon in marine sediments is mineralized via sulfate reduction (JØrgensen and Fenchel, 1974; Jørgensen, 1977, 1982). Despite its suggested antiquity, dissimilatory sulfate reduction (DSR) is patchily distributed among *Archaea* and *Bacteria* and has been experimentally observed solely within isolated members of four bacterial (*Deltaproteobacteria*, *Nitrospirae*, *Firmicutes*, and *Thermodesulfobacteria*) and two archaeal (*Euryarchaeota* and *Crenarchaeota*) lineages (Rabus et al., 2013). However, *dsrAB* genes have recently been detected in metagenomic assembled genomes (MAGs) affiliated with members of the phyla *Acidobacteria*, *Chloroflexi*, and several other phylum-level taxa, suggesting the capacity to perform DSR by some members of these phyla (Anantharaman et al., 2016, 2018; Hausmann et al., 2018).

In all recognized SRM, the reduction of sulfate to sulfide is mediated by three enzymes. In the first step of the process, ATP sulfurylase (Sat) activates chemically refractory sulfate to adenosine-5′-phosphosulfate (APS). The second enzyme, APS reductase (Apr, encoded by *aprBA*), reductively cleaves APS to AMP and bisulfite. The third enzyme, dissimilatory (bi)sulfite reductase (Dsr), reduces bisulfite to a protein-based trisulfide, in which the bisulfite-derived sulfur is bound to two cysteines of DsrC, the cellular levels of which can be used for determination of physiological sulfate reduction rates (Santos et al., 2015). The Dsr-encoding genes *dsrAB* are often used as the diagnostic enzyme for sulfate reduction in environmental studies (Rabus et al., 2013). Sulfate reduction is coupled with the oxidation of menaquinol in the membrane, which leads to the generation of a proton gradient via the membrane-bound redox complexes. Qmo (quinone-interacting membrane-bound oxidoreductase, encoded by *qmoABC*) transfers electrons to Apr, and the membrane-bound enzyme complex DsrMKJOP, which is homologous to HmeABCDE [heterodisulfide reductase (Hdr)-like, menaquinol-oxidizing enzyme], transfers electrons to Dsr during reduction of (bi)sulfite to sulfide (Santos et al., 2015; Dahl, 2017).

Proteins homologous to enzymes of the DSR pathway are also present and highly conserved in anoxygenic photolithotrophic and chemolithotrophic sulfur-oxidizing microorganisms (SOM), including, the strictly anaerobic, chlorophototrophic green sulfur bacteria (GSB) from the phylum *Chlorobi* (Dahl, 2017). Because these genes are used in both types of dissimilatory sulfur metabolism, the oxidative or reductive directionality of the pathway cannot easily be deduced from the presence of these genes alone. However, phylogenies for Dsr-encoding *dsrAB* and 16S rRNA genes are largely congruent with clear and distinct clades for SRM and SOM. Observed exceptions are mostly indicative of horizontal gene transfer (HGT) of *dsrAB* among major SRM taxa. Thus, the *dsrAB* genes are suitable diagnostic, phylogenetic, and functional marker genes (Klein et al., 2001; Zverlov et al., 2005; Loy et al., 2009; Müller et al., 2015; Pelikan et al., 2016; Hausmann et al., 2018), and have frequently been used as phylogenetic marker genes in amplicon-based sequencing in environmental studies (Dhillon et al., 2003; Nakagawa et al., 2004; Leloup et al., 2006; Dillon et al., 2007; Loy et al., 2009; Moreau et al., 2010; Mori et al., 2010; Pester et al., 2010; Lenk et al., 2011). However, it was recently shown that DsrAB phylogeny may be insufficient to distinguish the pathway directionality of sulfate reduction/sulfide oxidation in SRM and SOM, respectively. For example, *Desulfurivibrio alkaliphilus*, a deltaproteobacterium, is a SOM, but its DsrAB sequences are phylogenetically closer to those of SRM (Thorup et al., 2017).

The phylum *Chlorobi* has long been considered to be synonymous with its phototrophic, sulfur-oxidizing members, the GSB. The recent isolation and characterization of two chemoheterotrophic relatives, *Ignavibacterium album* and *Melioribacter roseus*, led to the restriction of the GSB to the family *Chlorobiaceae* within the class *Chlorobea*, one of six lineages within the *Chlorobi* (Iino et al., 2010; also see Liu et al., 2012a,b). The taxonomic level for these lineages has not been conclusively resolved. They were initially introduced as class-level lineages (Iino et al., 2010), but phylum level lineages have subsequently been suggested for some of them (e.g., *Ignavibacteriae* and “*Ca.* Kapabacteria,” but also *Chlorobaeota*, Podosokorskaya et al., 2013; Kantor et al., 2015; Oren et al., 2015). Sulfur oxidation in GSB relies on DSR genes similar to the ones found in SRM and other SOB. However, with regard to their DSR genes, GSB hold a special position and seem to contain a chimeric DSR system, in which some genes are most closely related to those of SOM, while others resemble those of SRM. It thus has been proposed that GSB have acquired either their whole or partial DSR system horizontally, although it is not clear whether the genes came from a SOM, a SRM, or both (Sander et al., 2006).

Microbial mats in the effluent channels of Octopus Spring and Mushroom Spring (MS), which are geochemically similar alkaline, siliceous hot springs in the Lower Geyser Basin of Yellowstone National Park (YNP), United States, are among the most intensively studied natural microbial communities (Ward et al., 2006). These mat communities have been studied for over 50 years and serve as model systems for exploring principles of microbial ecology (Brock, 1967; Ward et al., 1998; Ward et al., 2008). Most studies have focused on the numerous chlorophototrophic bacterial populations that occur in these mats (including *Cyanobacteria*, *Chloroflexi*, and the newly discovered species *Chloracidobacterium thermophilum* and “*Candidatus* Thermochlorobacter aerophilum” (Bryant et al., 2007; Liu et al., 2012b; Tank et al., 2017). Next-generation sequencing and metagenomic analyses were recently applied to characterize chemotrophic members of the community (Thiel et al., 2016, 2017). Although sulfate levels in the MS microbial mat and the spring water are low (<200 μM), the mat community sustains a highly active sulfur cycle (McCleskey et al., 2004; Dillon et al., 2007; Thiel et al., 2017). Previous cloning experiments targeting the *dsrAB* genes revealed four putative SRM in these mats: a *Thermodesulfovibrio* sp. (*Nitrospira*) and three additional unidentified phylotypes (Dillon et al., 2007).

Here, we describe a putative SRM that was initially discovered in MS and that is also found in other hot springs. We describe three partial genomes associated with non-chlorophototrophic organisms affiliated with “*Chlorobi*-lineage 5” (also known as “OPB56 clade” and “*Ca.* Kapabacteria”) (Kantor et al., 2015; Hiras et al., 2016). These organisms were identified by metagenomic analysis of hot spring microbial mat communities, and they represent the first SRM (or the first sulfur-oxidizing chemolithotroph) from the *Bacteroidetes*/*Chlorobi* group. The presence and diel transcription patterns of the genes strongly argue that the corresponding gene products are functional in these organisms. These findings raise important questions concerning the evolution of dissimilatory sulfur metabolism, the timing of the acquisition of these *dsr* genes within ancestral members of the *Chlorobi*, and the physiological flexibility of these novel organisms. Finally, these newly discovered, sulfur-metabolizing members of the *Bacteroidetes*/*Chlorobi* group may help to clarify the evolutionary origin(s) of dissimilatory sulfur oxidation within the GSB.

## Materials and Methods

### Sample Collection

**FIGURE 1:**
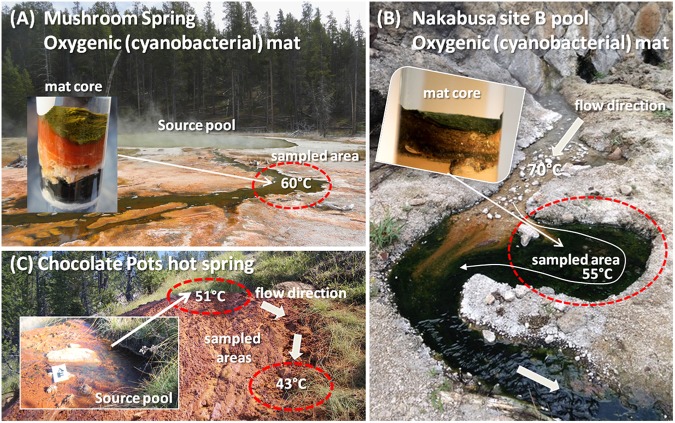
Photographs of hot springs and sampling sites in which the novel organism has been detected. **(A)** Mushroom Spring at YNP, WY, United States; **(B)** Nakabusa hot spring, site B pool in Nagano Prefecture, Japan; **(C)** Chocolate Pots hot spring at YNP, WY, United States. Insets in **A** and **B** show representative cores from the mats. Inset in **C** shows a close-up of the hot spring vent pool. Sampled areas are circled in red.

The samples analyzed in this study came from three different hot springs: MS, Chocolate Pots (both in YNP, WY, United States), and Nakabusa hot springs (Nagano Prefecture, Japan) (Figure 1). MS and Nakabusa hot springs are slightly alkaline (pH 8.0 and ∼8.5, respectively), while Chocolate Pots has a circumneutral pH (Fortney et al., 2016; Tank et al., 2017; Nishihara et al., 2018). Sulfate concentrations are relatively low in all three hot springs: Chocolate Pots, 26 μM; Nakabusa hot springs, 20 μM; and MS, <200 μM (McCleskey et al., 2004; Dillon et al., 2007; Fortney et al., 2016; Nishihara et al., 2018). Sulfide (46–138 μM) was only detected in Nakabusa hot springs source waters (Nishihara et al., 2018).

Mushroom Spring samples were collected from an oxygenic phototrophic microbial mat in one of the effluent channels of the slightly alkaline, siliceous spring at 60°C water temperature (GPS position 44.539/-110.798; Figure 1A) as previously described (Thiel et al., 2016, 2017). Samples for metatranscriptome analysis were taken at 19 different time points over the course of 1 day in 2014 (October 6, 13:00 to October 7, 17:00). Samples were taken every 1–3 h, and four biological replicates were collected. The microbial mat consists of a 1–2 mm upper green layer, which includes many types of chlorophototrophic bacteria (Tank et al., 2017) and an orange-colored undermat layer that is metabolically active to a depth of approximately 5–7 mm below the surface. Metagenomic, as well as partial metatranscriptomic analyses, of the chlorophototrophic community of the upper green layer have previously been published (Klatt et al., 2011, 2013; Liu et al., 2011; Thiel et al., 2016, 2017). In this study, the two layers were divided and separately analyzed.

Samples from Nakabusa hot springs were collected from an oxygenic, chlorophototrophic microbial mat from a small side pool that is fed by a stream of slightly alkaline spring water with a temperature of ∼55°C (Figure 1B). Triplicate samples, taken from the so-called “Stream Site” = “Site B” (36.393/-137.748, Nishihara et al., 2018), were collected from the region illustrated in Figure 1B over the time course of sampling. Samples for DNA extraction were collected in triplicate at the beginning and the end of a 39-h diel metatranscriptome experiment on November 3rd and 4th, 2016 using a #4 cork borer (ø = 8 mm). Samples for RNA extraction were collected hourly during the diel experiment in 2016 (November 3, 5:00 to November 6, 19:00, three biological replicates). The uppermost 3-mm mat layer, consisting of approximately equal parts of green and orange color, was separated from the rest using a knife, and the samples were quickly frozen in a dry-ice cooled, 70% (v/v) ethanol bath in 2-ml screw cap tubes. The samples were kept frozen on dry ice and were then transferred to a -80°C freezer until used. RNA from 20 of the 39 time points was sequenced for the metatranscriptomic analysis.

Iron-silica oxide samples were collected from two locations at Chocolate Pots (GPS position 44.710/-110.741; Figure 1C), a Fe-rich circumneutral-pH thermal spring, in September 2012 and used to initiate two microbial Fe(III)-reducing enrichment cultures, hereafter referred to as “vent” and “midway.” Temperature and pH at the two sample locations were 50.7°C, pH 5.9, and 42.8°C, pH 7.2 for the hot spring vent and midway down the flow path, respectively (Fortney et al., 2016). Fe(III)-reducing enrichment cultures were incubated in artificial spring water media following the geochemical measurements made by Parenteau and Cady (2010), at pH 6.1, 45°C, and pH 6.7, 40°C for the vent and midway enrichment cultures, respectively. Electron donor in the form of 2 mM acetate and 2 mM lactate was added to the media, and 10% (v/v) transfer to new media was made every 2 weeks, hereafter referred to as a generation (for details see Fortney et al., 2016).

### DNA Extraction

Genomic DNA was isolated from five replicate mat samples from MS using an enzymatic cell lysis protocol followed by phenol extraction as previously described (Thiel et al., 2016). For samples from Nakabusa hot springs, DNA was extracted and pooled from six replicate samples (three taken before and three after the diel experiment) using the MoBio PowerBiofilm DNA extraction kit (QIAGEN Inc., United States), following the manufacturer’s protocol. All centrifugation procedures were performed at 13,000 × *g* at room temperature. DNA concentration and purity was tested using dsDNA Broadrange (BR) assay on a Qubit fluorometer (Life Technologies, Grand Island, NY, United States) and Bio-Spec Nano (UV–VIS Spectrophotometer, Shimadzu, Japan).

Genomic DNA was extracted from generation 33 of the Chocolate Pots vent and midway Fe(III)-reducing enrichment cultures using the MoBio PowerSoil DNA isolation kit as previously described (Fortney et al., 2016).

### RNA Extraction

RNA from MS microbial mat samples was extracted using a hot-phenol extraction protocol adapted from Steunou et al. (2006) and Aiba et al. (1981). In brief, frozen mat samples were homogenized for 10 s using 0.5 g 150–200 μm glass beads in acidic acetate-EDTA buffer (250 μl 10 mM Na-acetate, pH 4.5; 37.5 μl 500 mM Na_2_-EDTA, pH 8.0). After the addition of 375 μl of lysis buffer [16 mM Na-acetate, 2% (w/v) SDS, pH 4.5], the sample was vortexed and incubated at 65°C for 3 min. Another 3-min incubation step followed at 65°C in hot acidic phenol, pH 4.5 (700 μl), before the samples were quickly cooled on ice prior to the initiation of phase separation by centrifugation (2 min at 17,000 × *g*). The aqueous phase was collected, and nucleic acids were purified with phenol:chloroform (1:1 v/v) and chloroform steps; the RNA was then precipitated using 0.1 volume of 10 M LiCl + 2.5 volumes 100% ethanol at -20°C for 30 min. Precipitated RNA was collected by centrifugation at 4°C (17,000 × *g*, 60 min), washed with 80% (v/v) ethanol, dried in a vacuum concentrator at room temperature, and resuspended in RNase-free water. Extracted RNA was treated with DNase following the Joint Genome Institute (JGI) recommendations with DNase I (2 U per 10 μg RNA; Ambion AM222) and then purified and concentrated using the QIAGEN RNeasy^®^ MinElute^®^ kit.

RNA was extracted from mat samples (0.10–0.21 g wet weight) from Nakabusa hot spring after centrifugation for 1 min at 13,000 × *g* at room temperature to remove excess liquid. These samples were subjected to RNA extraction using the RNeasy PowerBiofilm Kit (QIAGEN, Inc., United States) following the protocol of the manufacturer. The RNA was treated with DNase I, eluted in RNAse-free water (50 μl), and its concentration and purity were evaluated using the RNA High Sensitivity (HS) assay with Qubit fluorometer (Life Technologies, Grand Island, NY, United States) and Bio-Spec Nano (UV–VIS Spectrophotometer, Shimadzu, Japan).

### Sequencing

Genomic DNA extracted from MS mat samples was sequenced at the DOE JGI, United States, using Illumina HiSeq technology. Metagenome and 16S rRNA gene amplicon sequencing was conducted on Illumina HiSeq and MiSeq instruments, respectively, as described previously (Thiel et al., 2016, 2017). Genomic DNA extracted from Nakabusa hot spring microbial mat samples was sequenced using an Illumina MiSeq platform using a single run to obtain 2 × 300 paired-end reads at Fasmac (Atsugi, Japan). Purified DNA (300 ng) was used for library construction using the KAPA HyperPlus Library Prep Kit for Illumina (KAPA Biosystems) according to the protocol of the manufacturer.

Genomic DNA from the Chocolate Pots enrichment cultures was sequenced at the University of Wisconsin Biotechnology Center (UWBC^1^) using paired-end 2 × 250 bp Illumina MiSeq shotgun metagenomic sequencing technology.

Extracted total RNA samples from both microbial mats (MS and Nakabusa) were treated to remove rRNA using the TruSeq Ribo-Zero Bacteria kit (Illumina). The mRNA-enriched RNA was sequenced at JGI (MS samples) or DNALink Inc. (Seoul, South Korea, Nakabusa hot springs) using Illumina HiSeq 2000 and Next Seq500 instruments, respectively.

### Sequence Data Analyses

Metagenomic DNA sequence data from MS were analyzed as previously described (Thiel et al., 2016, 2017). Metagenomic data obtained from Nakabusa hot spring samples were analyzed as follows. Raw reads were trimmed, low-quality reads were removed using sickle tools ver. 1.33, and remaining reads were assembled using the SPAdes Genome Assembler ver. 3.7.1. after error correction using “BayesHammer” (Nikolenko et al., 2013). Assembly was conducted using the following kmers, *k* = 21, 33, 55, 77, 99, 127, with mismatch correction option “—careful.” Metagenome annotation was done using Prokka (Seemann, 2014). Metagenomic sequence data from the Chocolate Pots enrichment cultures were assembled using CLC Genomics Workbench 6.0.2^2^ as previously described (Fortney et al., 2016).

RNA samples isolated from MS collected over a diel cycle were sequenced on an Illumina HiSeq instrument at the JGI, United States. The raw data were pre-processed, quality checked, and trimmed as described for metagenome sequencing reads (Thiel et al., 2016). For a selection of 20 time-point samples from the Nakabusa diel experiment, RNA was sequenced using an Illumina Next Seq500 instrument. The raw RNA reads obtained were pre-processed using FastQC^3^. Adapter and low-quality reads were trimmed by Cutadapt version 1.12 (Martin, 2011). Quality-checked reads were mapped to the assembled reference metagenome using bowtie2 version 2.3.0 (Langmead and Salzberg, 2012) using the default settings allowing no mismatches, and aligned using the EDGE-pro algorithm (Magoc et al., 2013), using the rRNA depletion option. Differential expression analysis was conducted with DESeq2 (Love et al., 2014^4^).

### Binning, Annotation, and Phylogenetic Affiliations Using Marker Genes

Metagenomic contigs of the metagenomes obtained from MS and Nakabusa were binned on the basis of tetranucleotide frequency patterns, and the bins were visualized using emergent self-organizing maps (ESOM) as described (Thiel et al., 2017). Metagenomic contigs from the Chocolate Pots vent and midway metagenomic assemblies were binned using CONCOCT (Alneberg et al., 2014), and the taxonomic identities of the vent and midway MAGs were determined using PhyloSift (Darling et al., 2014). Unbinned vent and midway metagenomic assemblies were uploaded to Integrated Microbial Genomes (IMG) with Microbiome Expert Reviewer (IMG/M ER^5^) for gene annotation using the default IMG gene-calling method (Mavromatis et al., 2009). The scaffolds obtained from assembly and binning of the metagenomic sequences were treated as a single genome and automatically annotated using the RAST annotation server (Rapid Annotation using SEED Technology) (Aziz et al., 2008; Overbeek et al., 2014). Completeness and purity of MAGs were assessed using CheckM (Parks et al., 2015).

Phylogenetic affiliations were evaluated using the AMPHORA2 metagenomic workflow suite, which uses 31 universal bacterial phylogenetic marker genes for taxonomic affiliation of a genome sequence. This suite also uses Phyla_AMPHORA, which searches (partial) genomes for phylum-level, specific phylogenetic marker genes of 20 different bacterial phyla (Wu and Scott, 2012; Wang and Wu, 2013).

A set of 1544 representative, complete prokaryotic genomes was downloaded from the NCBI database^6^ [Filter settings “prokaryotes” (organism group), “Representative” (RefSeq category), “Complete genome” (Assembly level); accessed on March 15, 2018]. Phylogenetic marker genes were extracted from the genome sequences using AMPHORA2 (Wu and Scott, 2012). Genomes were filtered by the presence and number of phylogenetic marker genes detected, and a selection of 1152 representative prokaryotic genomes containing one copy of each marker gene per genome was used for further analysis. Additional partial genomes closely related to the metagenomic bins analyzed in this study were selected for analyses based on BLAST results of phylogenetic marker gene sequences. A total of 1171 (partial) genomes were used for phylogenetic analysis of phylogenetic marker genes.

Phylogenetic marker genes were extracted using AMPHORA2 from the (partial) genomes, and each set of extracted amino acid sequences was aligned using MEGA7 (Kumar et al., 2016) (Linux command line version M7CC, implemented MUSCLE align, data type “Protein,” standard settings except for “Gap Open” penalty value of -2.00 and “Gap Extend” penalty of -0.50, output format “FASTA”). The aligned sequences of 30 of the 31 marker genes (*smpB* was omitted due to low coverage) were concatenated using “Java Sequence Matrix”^7^. The output Nexus file was converted into FASTA format using AliView (Larsson, 2014). Alignments were imported into ARB (Ludwig et al., 2004) and manually refined. The amino acid alignment was filtered to exclude unalignable, highly variable regions within and between genes and was exported for phylogenetic analysis conducted using MEGA7. The maximum-likelihood phylogenetic analysis was based on the WAG model, with Gamma distributed rates among sites (five categories) using all sites selection. All non-mentioned parameters were used in default setting. Robustness of the trees was tested using non-parametric bootstrap analysis with 100 replicates in all cases.

Phylogenetic affiliations of MAGs and reference genomes were tested based on 16S rRNA gene sequences, when available. Phylogenetic trees were conducted based on the maximum-likelihood method using the “PhyML (DNA)” tool implemented in the software package ARB^8^ (Ludwig et al., 2004). Alignments of sequences obtained in this study as well as from database references were manually refined based on the SILVA/ARB SSU RefNR99 database Release 123^9^. GTR substitution model, four substitution rate categories, ML-based frequency estimates, as well as estimated ts/tv ration, gamma distribution parameter, and proportion of invariable sites settings were used. Robustness of branching was tested using 100 bootstrap replicates.

### Phylogenetic Analysis of *dsr* Genes

Based on their amino acid sequences, all genes related to DSR or oxidation were analyzed using Basic Local Alignment Tool (BLAST; Altschul et al., 1990) and conserved domain database (CDD) (Marchler-Bauer et al., 2015) available through the National Center for Biotechnology Information (NCBI), Bethesda, MD, United States. Phylogenetic analyses were conducted on concatenated sequences for DsrAB. Reference protein sequences for phylogenetic analyses were obtained by BLAST searches against a selected number of representative genome sequences in the JGI IMG system (Markowitz et al., 2014) and by BLAST searches against the NCBI nr database. The *dsrAB*/DsrAB genes/proteins were analyzed using the updated publicly available *dsrAB*/DsrAB database (Müller et al., 2015) and the ARB software package (Ludwig et al., 2004). Phylogenetic positions were determined by using the parsimony quick-add tool to add the DsrAB sequence to an existing phylogenetic tree. Using a number of reference sequences, a full maximum-likelihood tree was calculated using the PHYML (Amino Acids) tool implemented in ARB. Substitution model WAG, estimated gamma distribution, four substitution-rate categories, as well as estimated proportion of invariable sites and 100 bootstrap replicates were used.

### Transcriptomic Analyses

Using a proprietary mapping pipeline^10^, reads from the MS metatranscriptome were mapped onto the corresponding upper green mat layer and undermat metagenomes (IDs 3300010182 and 3300002493, respectively). Reads from the Nakabusa metatranscriptome were mapped onto the assembled Nakabusa metagenome using bowtie2 version 2.3.0, the default setting bowtie2 adding “–un-conc” option to collect “unmapped” reads. The read counts corresponding to mapped genes in the metagenome, which had been identified as belonging to bin MS-B_bin-24 (Thiel et al., 2017) (MS), and bin Naka2016_bin-10_OTU-51 (Nakabusa), were extracted with user-generated scripts and normalized for each time point as described (Liu et al., 2011, 2012b). Briefly, read counts were first normalized for each time point by the total number of read counts retrieved for the target organism at that time point. Secondly, the relative expression of each gene during the diel cycle was calculated by normalizing against the mean of all the reads at each time point for that particular gene. This method allows one to compare the relative transcript abundance levels, rather than absolute values, for each gene across the diel cycle. Genes were clustered according to their diel gene expression patterns using the k-means algorithm in Cluster 3.0 (Eisen et al., 1998), and results were visualized with Java Treeview (Saldanha, 2004) as described (Liu et al., 2012b). Normalized expression levels for each gene were imported into Cluster, adjusted by a log transformation, centered by mean, and then clustered using the k-means algorithm with *k* = 6, runs = 1000, and using other default parameters.

### Genome-Wide Sequence Comparison of MAGS

Taxonomic relationships between the three MAGs were tested by genome-wide average nucleotide identity (ANI) calculations using the online ANI calculator based on Goris et al. (2007)^11^.

### Data Availability

The YNP metagenomes are available at the JGI Genome Portal for IMG and Microbiomes, JGI IMG/MER^12^ with Taxon IDs 3300002493 (MS undermat metagenome) and 3300002895 (Chocolate Pots Midway enrichment). Metatranscriptome data for MS is available at the JGI with IMG Genome IDs3300005409, 3300005638, 3300006363, 3300006380, 3300010169, 3300010171, 3300010172, 3300010173, 3300010174, 3300010175, 3300010176, 3300010178, 3300010179, 3300010180, 3300010246, 3300010251, 3300010254, and 3300010256. MAGs are available from the NCBI database as WGS genomes under the accession numbers RCNO, RCNP, and RCNQ.****

## Results

### Metagenomic Data

Metagenomic sequencing and assembly of DNA from the undermat of MS (Figure 1) produced a 232-Mb metagenome comprising 315,154 total contigs (Thiel et al., 2017). The metagenome had a mean GC value of 54%, a maximum scaffold length of 158 kb, and a N/L50 value of 32,529/1.24 kb, which defines the number of fragments at or above the Length50 cutoff. There were 13,766 contigs >2.5 kb, which led to the recovery of 44 metagenomic bins (MAGs). Bin MS-B_bin-24 (Thiel et al., 2016, 2017) contained 2.6-Mb in a total of 109 contigs with a mean GC value of 38.5%. It contained a partial 23S rRNA gene, as well as 46 tRNA and 53 ribosomal protein genes (Table 1). Gene IDs for this MAG refer to the scaffold and gene IDs of the whole metagenome assembly, which is available at JGI IMG/MER (Taxon ID 3300002493). For convenience, the prefix of “JGI24185J35167_1” for each of the scaffold/gene IDs are omitted. Metagenomic sequencing of DNA from a hot-spring microbial mat from Nakabusa, Japan, resulted in 32,921,964 paired-end reads, which were assembled into a 320-Mb metagenome comprising 192,524 total contigs with a mean GC value of 54%, a maximum contig length of 1,260 kb, and a N50 value of 3,718 bp. Binning of 16,846 contigs ≥2.5 kb led to 76 MAGs; Naka2016_bin-10_OTU-51 contained 2.62 Mb in 32 contigs and importantly contained a complete rRNA operon (Table 1, and see below). Metagenomic sequencing and assembly of the DNA extracted from the Chocolate Pots vent and midway Fe(III)-reducing enrichment cultures yielded metagenomes of 42.1 Mb and 35,690 contigs, and 86.0 Mb and 93,531 contigs, respectively. The vent enrichment culture metagenomic assembly had a mean GC content of 54.1%, a maximum scaffold length of 657 kb, and an N50 value of 2,072. The midway metagenomic assembly had a mean GC content of 52.7%, a maximum scaffold length of 744 kb, and an N50 value of 1,149. The vent and midway enrichment culture metagenomic assemblies are available at JGI/MER under Taxon Object IDs 3300002900 and 3300002895, respectively. A total of 1,520 contigs >3 kb were binned into eight MAGs with an average read coverage of 16.2× in the vent enrichment culture metagenomic assembly. 2,148 contigs >3 kb were binned into 15 MAGs with an average read coverage of 13.4× in the midway assembly. A MAG “CP-midway-bin-8,” which was assembled from metagenomic sequences from the midway enrichment culture from Chocolate Pots thermal feature, YNP, had a total sequence length of 2.85 Mb, 33.8% GC content, and comprised 359 contigs with an average read coverage of 7.5×. Statistics for these three MAGs are provided in Table 1. Each of the three MAGs described above contained *dsrAB* genes, and these metagenomic bins representing putatively novel SRM/SOM organisms were analyzed further.

**Table 1:** Characteristics of MAGs obtained in this study and one reference genome obtained from the NCBI database.

		Size	Number of		NCBI WGS	Completeness	Contamination			Ribosomal
Bin name	Source	(Mb)	contigs	GC (%)		[%]	[%]	rRNA	tRNA	proteins
MS-B_bin-24	Mushroom Spring, YNP, United States	2.60	109	38.5	RCNO00000000.1	90.6	3.3	LSU	46	53
Naka2016_bin-10	Nakabusa hot spring, Japan	2.62	32	37.1	RCNP00000000.1	95.8	0	SSU, LSU	46	56
ChocPot-Midway-bin-8	Chocolate Pots hot spring, YNP, United States	2.85	359	33.8	RCNQ00000000.1	87.7	1.4	None	42	48
Ignavibacteria bacterium GWB2_35_12	Groundwater aquifer at Rifle, CO, United States	4.02	200	35.0	MGZV00000000.1	96.3	0.6	5S, SSU, LSU	47	54


*Relative completeness and contamination were assessed using CheckM (Parks et al., 2015).*

### Phylogenetic Affiliation of the Three MAGs

#### Phylogenetic Analysis of Marker Genes

**FIGURE 2:**
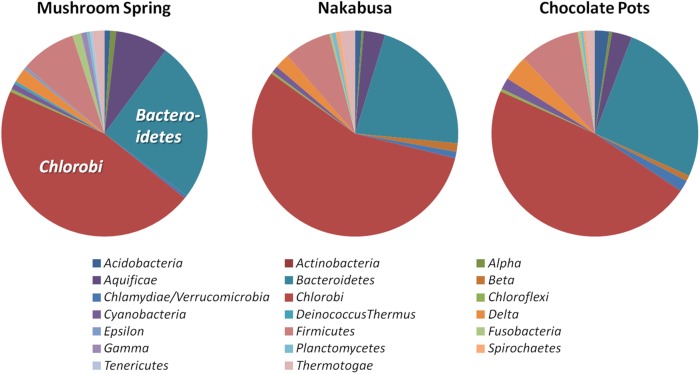
Taxonomic affiliations of metagenomic bins based on phylogenetic marker genes of 20 different bacterial phyla (Phyla_AMPHORA; Wang and Wu, 2013). The majority of phylogenetic marker genes detected in the partial genomes belonged to the taxa *Chlorobi* (100–167 of 808 phylum-specific marker genes) or *Bacteroidetes* (55–65 of 215 phylum-specific marker genes) (Wang and Wu, 2013). Detailed number of genes detected for each phylum can be found in Table 2.

**FIGURE 3:**
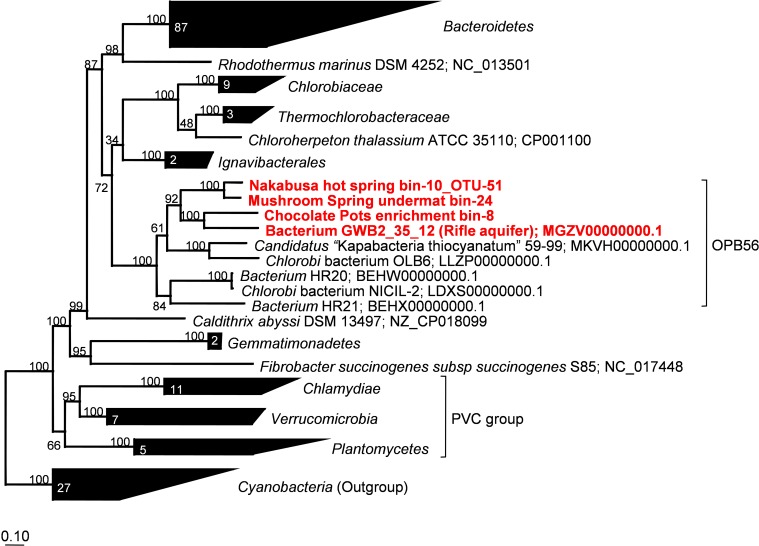
Maximum-likelihood tree based on 30 concatenated phylogenetic marker genes showing the phylogenetic affiliations of the metagenome bins analyzed in this study. Amino acid sequences extracted from 166 representative and partial genomes and a total of 3,187 sites were used for calculation of the tree using MEGA7 (Kumar et al., 2016). Robustness of the tree topology was tested with 100 bootstrap replicates. The scale bar represents 0.1 changes per amino acid position. Numbers in collapsed branches represent number of sequences in the clusters. PVC = Planctomycetes, Verrucomicrobia, Chlamydiae.

Phylogenetic marker-gene analysis by Amphoranet and Phyla_Amphora indicated that the three MAGs were taxonomically affiliated with the *Bacteroidetes*/*Chlorobi* group (Figures 2, 3 and Table 2). About half of the phylum-level, phylogenetic marker genes in each MAG were affiliated with *Chlorobi*, and a further 20% of the marker genes were affiliated with *Bacteroidetes*. The remaining marker genes were distributed among several phyla, with *Firmicutes* and *Aquificae* being the next most abundant (Figure 2). Specifically, the individual MAGs contained 100–167 of 808 phylum-level marker genes specific for *Chlorobi*; furthermore, the bins contained 55–65 of 215 *Bacteroidetes*-specific marker genes (Figure 2). By comparison, the genomes of *M. roseus* and *I. album* contain 184 and 185 *Chlorobi*-specific, and 73 and 76 *Bacteroidetes*-specific marker genes, respectively. A phylogenetic analysis of the concatenated amino acid sequences for 30 marker–gene products further supported the affiliation of the MAGs with the *Bacteroidetes*/*Chlorobi* superphylum (Figure 3).

**Table 2:** Number of identified phylogenetic marker genes for 20 different bacterial phyla in the three hot spring derived ‘*Ca*. Thermonerobacter spp.’ MAGs as well as the Rifle aquifer-derived database MAG GWB2_35_12 (WGS acc. no. MGZV01; genes identified by Phyla_AMPHORA, Wang and Wu, 2013).

Phylum	ChocPot_Midway_bin-8	MS-B_bin-24	Naka2016_bin-10	GWB2_35_12	Mean	*SD*
*Acidobacteria*	5	2	3	3	3.25	1.26
*Actinobacteria*	0	0	0	2	0.5	1.00
*^∗^Alpha*	1	2	1	1	1.25	0.50
*Aquificae*	7	18	10	4	9.75	6.02
*Bacteroidetes*	58	55	65	60	59.5	4.20
*^∗^Beta*	2	0	4	1	1.75	1.71
*Chlamydiae*/*Verrucomicrobia*	4	1	3	3	2.75	1.26
*Chlorobi*	106	100	167	138	127.75	31.03
*Chloroflexi*	1	1	1	0	0.75	0.50
*Cyanobacteria*	4	2	3	4	3.25	0.96
*Deinococcus–Thermus*	0	1	0	0	0.25	0.50
*^∗^Delta*	9	5	7	10	7.75	2.22
*^∗^Epsilon*	0	1	0	2	0.75	0.96
*Firmicutes*	21	19	22	17	19.75	2.22
*Fusobacteria*	1	3	1	1	1.5	1.00
*^∗^Gamma*	0	2	0	0	0.5	1.00
*Planctomycetes*	1	1	2	2	1.5	0.58
*Spirochaetes*	1	0	2	1	1	0.82
*Tenericutes*	0	1	0	0	0.25	0.50
*Thermotogae*	3	4	7	2	4	2.16
Total	224	218	298	251	247.75	36.45


*^∗^Alpha, Beta, Delta, Gamma, and Epsilon refer to classes within the Proteobacteria.*

#### Phylogenetic Affiliation Based on 16S rRNA

**FIGURE 4:**
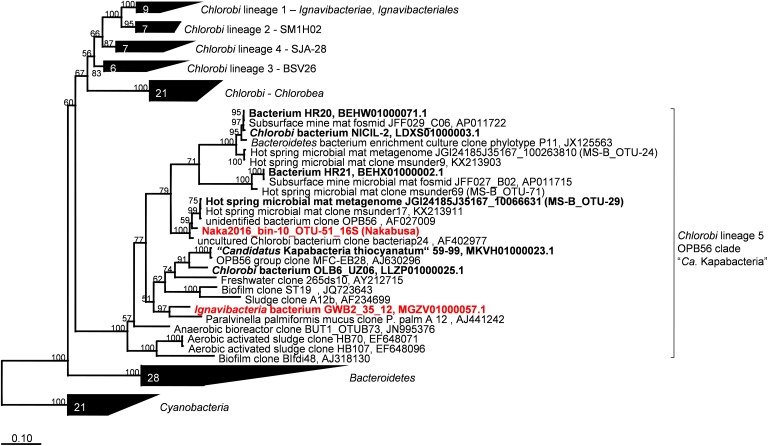
Maximum-likelihood tree of 16S rRNA gene sequences derived from the metagenome bins analyzed in this study and closely related partial genomes showing their phylogenetic affiliation with the “*Chlorobi* lineage 5” (OPB56 clade, “*Ca.* Kapabacteria”). Percentage numbers on nodes refer to 100 bootstrap pseudo-replicates conducted. Only values >50% are shown. Scale bar represents 0.1 changes per nucleotide position.

Metagenomic bin “Naka2016_bin-10_OTU-51” from Nakabusa hot spring contained a complete rRNA operon on contig_660. However, MS-B_bin-24 only contained a partial 23S rRNA gene, and the ChocPot-Midway-bin-8 did not contain any rRNA sequences (Table 1). The 23S rRNA gene sequences obtained from Mushroom and Nakabusa hot springs share 98% nucleotide identity, indicating a close relationship between the two organisms. They are additionally closely similar to *Bacteroides* and *Chlorobi* fosmid clones JFF029_C06 and JFF027_B02 (NCBI acc. nos. AP011722 and AP011715, 86–90% nt ID) as well as to the type strains of *I. album* and *M. roseus* (acc. nos. CP003418/CP003557, 85% nt identity). Phylogenetic analysis of the available 16S rRNA gene sequence placed the organisms represented by the hot-spring-derived MAGs into the “*Chlorobi*-lineage 5” (Hiras et al., 2016, also known as “OPB56 clade”; it has recently been suggested that the organisms in this group represent a novel phylum with the name “*Candidatus* Kapabacteria” Kantor et al., 2015) (Figure 4). A closely related 16S rRNA gene sequence (97% nt identity to the sequence from Nakabusa, MS-B_OTU-29, Thiel et al., 2016) was found in the metagenome produced from MS; it was too short to be assigned during binning but is also consistent with this assignment. All closely related reference genomes that cluster with the novel MAGs in the phylogenetic tree on the basis of the concatenated phylogenetic marker genes in Figure 3 were also placed within “*Chlorobi* lineage 5”—i.e., the OPB56 cluster on the basis of 16S rRNA sequences (Figure 4). This includes “*Ca.* Kapabacteria thiocyanatum” as well as organisms represented by MAGs obtained from bioreactors (Kantor et al., 2015), anammox wastewater treatment plants (Speth et al., 2016), compost (Hiras et al., 2016), and a microbial mat from a subsurface geothermal stream (Kato et al., 2018), respectively. Although the ribosomal genes indicate a close relationship between the organisms obtained from MS and Nakabusa, a genome wide comparison indicates an ANI between the two partial genomes of only 75%, which does not support the conclusion that these two organisms are the same species. Therefore, we propose the genus name “*Candidatus* Thermonerobacter” (Thermonerobacter, thermós, Greek for hot/thermal; nero, Greek for water; bacter, from bakterion, Greek for bacterium) for the novel organisms obtained from MS and Nakabusa hot springs microbial mats, and the species name “*Candidatus* Thermonerobacter thiotrophicus” (thiotrophicus, thio from Greek theion for sulfur; trophicus from Greek trophus, eater) for the organism inhabiting MS.

#### Sulfur Metabolism and Clustering of *dsr* Genes

Complete sets of genes for DSR (or sulfide oxidation) were present in two of the three partial genomes recovered from the hot-spring metagenomes. The slightly less complete metagenomic bin (ChocPot-Midway-bin-8) obtained from the Chocolate Pots enrichment culture was missing 2 (*dsrP* and *qmoA*) of the 20 required genes (Table 3).

**Table 3:** Dissimilatory sulfate-reduction (DSR)-associated genes detected in metagenome bins and partial genomes.

Gene	Enzyme	Mushroom	Nakabusa	Chocolate	Database
		Spring		Pot	(strain
					GWB)
*aprA*	Adenosine-5’-phosphosulfate reductase alpha subunit	Yes	Yes	Yes	Yes
*aprB*	Adenosine-5’-phosphosulfate reductase beta subunit	Yes	Yes	Yes	Yes
*dsrA*	Dissimilatory sulfite reductase (desulfoviridin), alpha subunit	Yes	Yes	Yes	Yes
*dsrB*	Dissimilatory sulfite reductase (desulfoviridin), beta subunit	Yes	Yes	Yes	Yes
*dsrC*	Dissimilatory sulfite reductase subunit C (DsrC)	Yes	Yes	Yes	Yes
*dsrD*	dissimilatory sulfite reductase subunit D (DsrD)	Yes	Yes	Yes	Yes
*dsrJ*	DSR system component, protein DsrJ/HmeE	Yes	Yes	Yes	Yes
*dsrK*	DSR system component, protein DsrK/HmeD	Yes	Yes	Yes	Yes
*dsrK-2*	DSR system component, protein DsrK/HmeD	Yes	Yes	Yes	Yes
*dsrL*	Iron-sulfur-binding protein, glutamate synthase (dsrL/gltD)	Yes	Yes	Yes	Yes
*dsrM*	DSR system component, protein DsrM/Hdr-like menaquinol-oxidizing enzyme, subunit C (HmeC/DsrM)	Yes	Yes	Yes	Yes
*dsrM-2*	DSR system component, protein DsrM/Hdr-like menaquinol-oxidizing enzyme, subunit C (HmeC/DsrM)	Yes	Yes	Yes	Yes
*dsrN*	cobyrinic acid A,C-diamide synthase, siroheme a-amid synthase (DsrN)	Yes	Yes	Yes	Yes
*dsrO*	DSR system component, protein DsrO/HmeA	Yes	Yes	Yes	Yes
*dsrP*	DSR system component, protein DsrP / HmeB	Yes	Yes	No	Yes
*dsrT*	dissimilative sulfite reductase protein dsrT / DSR system component, protein DsrT	Yes	Yes	Yes	Yes
*qmoA*	Quinone-interacting membrane-bound oxidoreductase complex subunit A	Yes	Yes	No	Yes
*qmoB*	Quinone-interacting membrane-bound oxidoreductase complex subunit B	Yes	Yes	Yes	Yes
*qmoC*	heterodisulfide reductuase / quinone-interactin membrane-bound oxidoreductase, subunit C	Yes	Yes	Yes	Yes
*sat*	ATP sulfurylase (sulfate adenylyltransferase)	Yes	Yes	Yes	Yes


*Database (strain GWB) refers to ‘Ignavibacteria bacterium GWB2_35_12,’ obtained from groundwater aquifer at Rifle, CO, United States; WGS entry MGZV00000000.1.*

**FIGURE 5:**
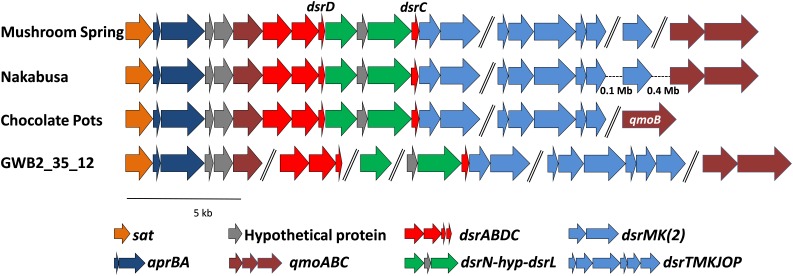
Organization of the genes related to DSR in the MAGs derived from MS, Nakabusa, and Chocolate Pots, as well as from the “*Ignavibacteriaceae* bacterium GWB2_25_12” obtained from a groundwater aquifer at Rifle, CO, United States (NCBI database WGS MGZV00000000.1). The scale bar indicates 5 kb, and all genes are shown at similar scale. Genes separated with “//” are located on different contigs, while hyphens, “-”, indicate gaps between genes located on the same contig.

The genes required for DSR were located on four (MS), two (Nakabusa), or three (Chocolate Pots) different contigs, respectively (Figure 5). The overall organization of the genes required for sulfide oxidation/sulfate reduction was identical for the three hot-spring MAGs. The genes encoding ATP sulfurylase (*sat*) and APS reductase (*aprBA*) occur in an apparent operon with genes encoding the membrane-bound QmoABC complex (*qmoABC*) in GSB (Frigaard and Bryant, 2008; Bryant et al., 2012). In the MAGs described here, the *sat* genes were always found adjacent to *aprBA*, but in contrast to GSB, *aprA* was upstream of two conserved hypothetical genes before *qmoC*, which encodes the membrane-bound, heme *b*-binding subunit of the heterodisulfide-reductase-like “quinone-interacting, membrane-bound oxidoreductase” (Qmo) complex (Figure 5). The *qmoA* and *qmoB* genes were adjacent in two of the three MAGs, but these genes were found in different chromosomal locations in the three MAGs (Figure 5). Although *qmoA* was not present in the Chocolate Pots metagenome, it probably occurs upstream of *qmoB*, which is located at the 5′- end of a contig.

The *dsrAB* genes are highly conserved components of the Dsr and rDsr (i.e., reversely operating Dsr found in sulfur-oxidizing bacteria) enzymes, which are responsible for the reduction of bisulfite to sulfide or the oxidation of sulfide to bisulfite, respectively (Frigaard and Dahl, 2008; Müller et al., 2015; Santos et al., 2015; Dahl, 2017). In the three hot-spring MAGs, the *sat-aprBA-hyp-hyp-qmoC* operon was followed by genes encoding the Dsr/rDsr enzyme. The *dsrAB* genes were followed by *dsrD-dsrN-hyp-dsrL-dsrC* and homologs of *dsrM* and *dsrK* (*dsrM-2* and *dsrK-*2 in Table 3 and Figure 5). The co-occurrence of *dsrD* and *dsrL* is unusual, and has only been found in a newly described and uncultured member of the phylum *Acidobacteria* with dissimilatory sulfur metabolism (Hausmann et al., 2018). Ubiquity of DsrD among SRM suggests an essential function in sulfate reduction, while DsrL usually has been associated with SOMs (Hittel and Voordouw, 2000; Lübbe et al., 2006; Weissgerber et al., 2014; Hausmann et al., 2018). However, since the functions of DsrL and DsrD are still unresolved, functional predictions based only on the presence of these genes are not possible. Genes encoding the presumed menaquinol-oxidizing enzyme complex (*dsrTMKJOP*), which encode subunits similar to those of membrane-bound heterodisulfide reductases, showed the same organizational pattern in the metagenomes of bins recovered from the microbial mat communities of Mushroom and Nakabusa hot springs. In both cases *dsrT* occurs upstream from *dsrMKJO*, but *dsrP* is located elsewhere in the genome (Figure 5). Although not originally annotated by RAST, a partial sequence for *dsrO* was found downstream from *dsrJ* on contig_2519 (Figure 5). The *dsrP* gene is assumed to be present elsewhere in the genome on a contig that was not included in the metagenomic bin.

#### *dsrAB* Identity and Phylogeny

**FIGURE 6:**
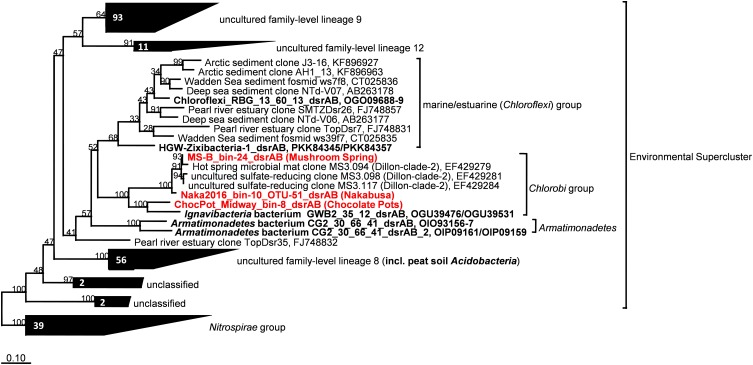
Maximum-likelihood tree based on DsrAB amino acid sequences displaying the phylogenetic affiliation of the novel thermophilic putative SRB *Bacteroidetes/Chlorobi* group member within the Environmental Supercluster. Sequences obtained in this study are indicated in *red bold font*. Sequences shown in *black bold font* were obtained from MAGs with known phylogenetic affiliations from previous studies. The tree was constructed using an alignment in the publicly available DsrAB ARB database (Müller et al., 2015; http://www.microbial-ecology.net/download). Robustness of branching patterns was tested with 100 bootstrap replicates. Scale bar represents 0.1 changes per amino acid position.

The *dsrAB* genes, which were detected in the bin MS-B_bin-24 metagenome (Table 1) and which encode the alpha and beta subunits of Dsr/rDsr, are most closely related to *dsrAB* clone sequences obtained from MS in a previous study (Dillon et al., 2007; Table 4 and Figure 6). The *dsrAB* sequences obtained from the MS metagenome share 98–99% nucleotide and 94–98% amino acid sequence identity to clones MS3.094, MS3.117, and MS3.098 (NCBI acc. nos. EF429279, EF429284, EF429281); these were identified as “clade 2” with unknown phylogenetic affiliation in a *dsrAB*-targeted, cloning study of the MS microbial mat community (Dillon et al., 2007). Those clones are also the best BLAST hits to *dsrAB* sequences derived from the Nakabusa hot-spring metagenome (Table 4). Furthermore, phylogenetic analysis of the *dsrAB* sequences from the three hot-spring MAGs were closely affiliated with other organisms with “unclassified environmental sequences” within the “environmental supercluster 1” of unidentified and uncultured reductive bacterial-(=sulfate-reducing bacteria)-type *dsrAB* clade (Müller et al., 2015; Figure 6 and Supplementary Figure S1). The sequences found in this study do not cluster with any of the recently published DsrAB sequences found in other MAGs, such as the peat soil members of the *Acidobacteria* (Hausmann et al., 2018).

**Table 4:** Next closest relatives of the putative SRB analyzed in this study as determined by DsrAB BLASTp search results (five best hits containing both DsrA and DsrB considered)

		*DsrA*				*DsrB*			
			
MAG	BLAST hit name	Hit accession	E value	Ident (%)	Pos (%)	Hit accession	E value	Ident (%)	Pos (%)
MS-B_bin-24	clone MS3.094 (MS, YNP)	AB021841	0	99	99	AB021842	0	100	100
	clone MS3.117 (MS, YNP)	AB021851	0	99	100	AB021852	3.9423E-160	100	100
	clone MS3.098 (MS, YNP)	AB021845	0	95	96	AB021846	0	99	99
	Ignavibacteria bacterium GWB2_35_12	OGU39476	0	77	89	OGU39531	0	78	78
	Armatimonadetes bacterium CG06_land_8_20_14_3_00_66_21	PIU88856	0	72	86	PIU88857	0	76	76

ChocPot_bin-8	Ignavibacteria bacterium GWB2_35_12	OGU39476	0	85	94	OGU39531	0	85	85
	Chloroflexi bacterium RBG_13_60_13	OGO09688	0	76	86	OGO09689	0	76	76
	Fosmid 39f7 (Wadden Sea)	CAJ31162	0	75	86	CAJ31163	0	75	75
	Candidate division Zixibacteria bacterium HGW-Zixibacteria-1	PKK84345	0	74	87	PKK84357	0	80	80
	Fosmid ws7f8 (Wadden Sea)	CAJ31214	0	74	87	CAJ 31213	0	75	75
	Ignavibacteria bacterium GWB2_35_12	OGU39476	0	78	89	OGU39531	0	77	77

Naka2016_bin-10	Clone MS3.117 (MS, YNP)	AB021851	0	95	99	AB021852	1.2003E-154	95	95
	Clone MS3.094 (MS, YNP)	AB021841	0	94	98	AB021842	1.34E-176	95	95
	Clone MS3.098 (MS, YNP)	AB021845	0	91	94	AB021846	2.3949E-176	95	95
	Armatimonadetes bacterium CG06_land_8_20_14_3_00_66_21	PIU88856	0	73	86	PIU88857	0	75	75

GWB2_35_12	Ignavibacteria bacterium GWB2_35_12	OGU39476	0	100	100	OGU39531	0	100	100
	Candidate division Zixibacteria bacterium HGW-Zixibacteria-1	PKK84345	0	74	88	PKK84357	0	80	80
	Chloroflexi bacterium RBG_13_60_13	OGO09688	0	74	84	OGO09689	0	75	75
	Fosmid 39f7 (Wadden Sea)	CAJ31162	0	73	87	CAJ31163	0	75	75
	Fosmid ws7f8 (Wadden Sea)	CAJ31214	0	73	85	CAJ 31213	0	73	73


#### Additional Genes Possibly Affiliated With Sulfur Metabolism

In addition to the 20 genes required for DSR (or sulfide oxidation), several other genes related to sulfur metabolism were found in the bins. Three genes encoding a membrane-bound molybdopterin oxidoreductase of the Psr/Psh family (MS gene IDs 01959_007-009) were present in the MS and Nakabusa MAGs. These genes are most similar to the *psrABC* genes found in *M. roseus* [MROS_1774 to 1776] and *I. album* [IALB_1661 to 1663] (60–78% aa identity). The subunits of this oxidoreductase are similar to the corresponding components of polysulfide and nitrite reductases, and they show 37–52% amino acid sequence identity to polysulfide reductase-like complex 2 (PSRLC2) in GSB (Hinsley and Berks, 2002; Liu et al., 2012a; Kadnikov et al., 2013). A putative transcriptional regulator of the MerR family (MS gene ID 01959_010) precedes these three genes in the MS and Nakabusa MAGs. Transcription patterns (see below) as well as nearby genes located downstream of *psrABC* support a function in sulfur metabolism: (1) a putative sulfur transporter of the DUF395 family containing two sulfur transport domains (MS gene ID 01959_005/006 and Pfam family Sulf_transp PF04143) and (2) a rhodanese-related sulfurtransferase (MS gene ID 01959_004). Sulfur transporter and sulfurtransferase genes are also present in the Nakabusa MAG, notably on the same contig and only a few genes downstream of the DSR operon. Homologs of PSRLC3, an alternative sulfite-oxidizing system found in GSB, were not found in the metagenomic sequences (Frigaard and Dahl, 2008). Finally, no sulfide:quinone reductases (*sqr*) were detected in the partial genomes. This is in contrast to GSB but is nevertheless consistent with the idea that these members of the *Bacteriodetes*/*Chlorobi* group reduce sulfate rather than oxidize sulfide. Interestingly, the *I. album* genome encodes two sulfide:quinone reductase genes but lacks the genes for sulfate reduction (Liu et al., 2012a).

### Central Carbohydrate Metabolism and Carbon Dioxide Fixation

**FIGURE 7:**
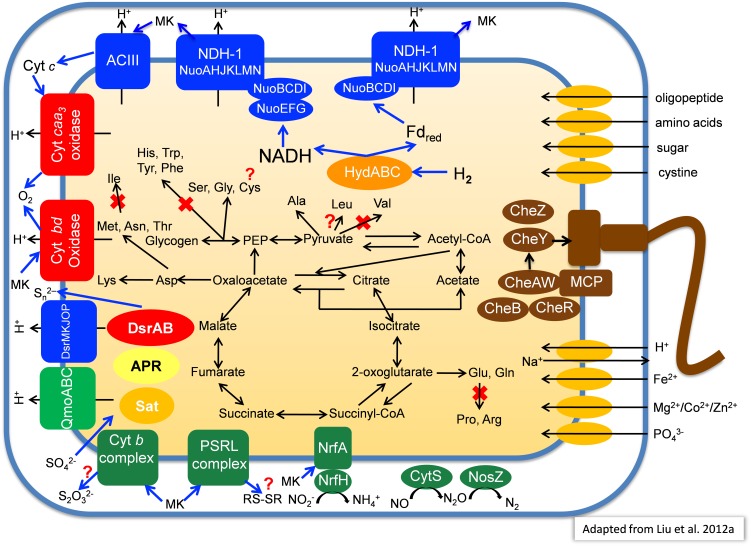
Metabolic model of “*Candidatus* Thermonerobacter thiotrophicus” showing selected pathways. For additional details, see text. Red X’s indicate pathways that based on the current information are most likely not present in “*Ca*. T. thiotrophicus,” while question marks indicate incomplete but putatively functional, or otherwise uncertain pathways.

Primarily based on the gene content of the MAG obtained from MS, Figure 7 summarizes a number of key aspects of the anticipated physiology and metabolism for the putative SRM. These uncultured organisms include a complete set of genes for glycolysis and the TCA cycle which suggests that they have the ability to oxidize organic substrates completely to CO_2_.

Many SRM can use the Wood–Ljungdahl pathway (Fuchs, 2011), including carbon monoxide dehydrogenase (EC 1.2.7.4)/CO-methylating acetyl CoA synthase (acetyl-CoA synthase, EC 2.3.1.169), to synthesize acetate from CO_2_. However, the genes for this key enzyme were absent from all three MAGs. Likewise, genes for other CO_2_ fixation pathways were not present in the MAGs, which implies that these organisms cannot grow autotrophically. Nevertheless, genes for pyruvate:ferredoxin oxidoreductase (PFOR, pyruvate synthase; EC 1.2.7, gene ID 000011_21) and 2-oxoglutarate:ferredoxin oxidoreductase (KFOR EC 1.2.7.3; gene ID 000227_19-20), which can carboxylate acetyl-CoA and succinyl-CoA, respectively, were present in the metagenomes (see description of the section “Amino Acid Metabolism”). Thus, these organisms might incorporate some CO_2_ into biomass as apparently occurs for *C. thermophilum* (Tank and Bryant, 2015a,b). The presence of acetyl-CoA synthetase (gene ID 000018_23, EC 6.2.1.1) further suggests these organisms can probably assimilate acetate. Finally, the presence of genes for phosphate acetyltransferase (phosphotransacetylase, EC 2.3.1.8; gene ID 000091_20) and acetate kinase (EC 2.7.2.1, gene ID 000091_21) suggests that under some conditions, these organisms may use acetyl-phosphate to produce ATP by substrate-level phosphorylation with subsequent excretion of acetate.

### Electron Transfer and Respiration

Similar to the genome of *I. album* (Liu et al., 2012a), the three hot-spring MAGs contain two sets of genes encoding type-I NADH dehydrogenase: one set encodes 14 subunits (*nuoABCDEFGHIJKLMN*) that are present in different parts of the genome, while the other set lacks the *nuoEFG* genes and comprises a single 11-gene operon (Figure 7). The absence of *nuoC* in the metagenomic bin derived from Chocolate Pots is assumed to be due to incomplete genome coverage. Genes closely related to NuoEFG (or to hydrogenase subunits, HydABC) are encoded elsewhere in the genome and appear to encode a soluble, bifurcating hydrogenase (Schut and Adams, 2009). Such an enzyme could reversibly oxidize hydrogen and produce NADH and reduced ferredoxin as products. These products could be re-oxidized by the two type-1 NADH dehydrogenase complexes as shown in Figure 7. If the NADH dehydrogenase complex and bifurcating Hyd act together, up to four protons could be translocated per hydrogen molecule oxidized because both NADH and reduced ferredoxin can be used to reduce quinones. As such, this mechanism would be more favorable than using membrane-bound hydrogenases to oxidize hydrogen and directly produce reduced quinones, which would account for only two protons translocated (Buckel and Thauer, 2018a,b).

Three genes encoding succinate dehydrogenase (complex II) as well as five genes encoding alternative complex III (ACIII) are present in each of the four partial genomes. The identified genes for the latter share highest sequence similarities to corresponding *act* genes of *I. album* (IALB_1386, 1388, 1389, 1390, and 1391) and *M. roseus* (MROS_0041-45). Similar to *Dokdonia* sp. MED134, *Schleiferia* sp., and other members of the *Bacteroidetes*, as well as *Bacterioidetes*/*Chlorobi* members *I. album* and *M. roseus*, genes encoding ubiquinol:cytochrome *c* oxidoreductase (complex III) were not detected (González et al., 2011; Liu et al., 2012a; Kadnikov et al., 2013; Thiel et al., 2014).

Four genes (MS gene ID 000332_3-6) encoding an *aa*_3_-type cytochrome *c* oxidase (complex IV) are present in each of the MAGs, which suggests these putative SRM have the ability to perform aerobic respiration for growth under some conditions or can detoxify and tolerate relatively high oxygen concentrations. Additionally, genes for a cytochrome *bd*-type quinol oxidase are present; this type of quinol oxidase typically has a very high affinity for oxygen, and thus it can function efficiently at very low oxygen concentrations (Forte et al., 2017). In contrast to the genomes of *I. album*, *M. roseus*, and some GSB strains, genes encoding an alternative, high-affinity cytochrome *cbb*_3_-type oxidase were not observed in the four partial genomes. As found for *I. album*, a complete Na^+^-translocating Fd:NAD^+^ oxidoreductase (RNF complex) was present in the partial genome from Nakabusa. RnfCDEGAB-encoding genes most closely related to genes in *Ignavibacteriaceae* and the Nakabusa sequences were also detected in the MS metagenome on several short contigs not included in the binning. This might suggest that the RNF complex also occurs in the organism found in MS, but at the moment this cannot be inferred conclusively (Thiel et al., 2016).

In addition to possible aerobic respiration and DSR, genes encoding other terminal reductases of anaerobic respiratory pathways are present in the analyzed MAGs. Although no genes for nitrate reductase were found in any of the MAGs, all three contain NrfAH-type nitrite reductase (MS gene ID 00001139-40), nitric oxide reductase (CytS, MS gene ID 000001125), and nitrous oxide (NosZ, MS gene ID 00000354) reductase (Figure 7). This is similar to the situation in *M. roseus* (Kadnikov et al., 2013).

### Motility

Genes necessary for flagellar motility were present in the metagenomes, but the flagellar genes do not form a single gene cluster as in the *I. album* genome. Furthermore, a complete set of chemotaxis proteins, CheABWRYZ were found in the MS and Nakabusa MAGs, although only some genes were initially affiliated with MS-B_bin-24 while others were found on contigs too short to be reliably binned. Currently, the identities of potential attractants and/or repellants are unknown.

### Amino Acid Metabolism

On the basis of the partial genomic information, a complete pathway for lysine biosynthesis is present; this is similar to *Chloroherpeton thalassium* but unlike *I. album*, which lacks this ability. Only incomplete metabolic pathway information for biosynthesis of other amino acids exists at present. However, based on the information currently available in the partial genomes, these organisms probably are unable to synthesize two branched-chain amino acids, valine and isoleucine. In contrast, the ability to synthesize leucine is assumed based on the presence of all genes needed from the intermediate 2-isopropylmalate, although 2-isopropylmalate synthase (EC 2.3.3.13) has not been detected. Notably, they should be able to degrade all branched-chain amino acids like “*Ca.* T. aerophilum,” and *C. thermophilum* (Liu et al., 2012b; Tank and Bryant, 2015a,b). Thus, these organisms may primarily synthesize 2-oxoglutarate by carboxylation of succinyl-CoA as has been suggested to occur in *C. thermophilum* and “*Ca.* T. aerophilum” (Liu et al., 2012b; Tank and Bryant, 2015a,b; Tank et al., 2017). The currently available information from the three metagenomes suggests that these SRM may be unable to synthesize aromatic amino acids (phenylalanine, tyrosine, tryptophan), histidine, arginine, proline, and possibly cysteine, while serine, glycine, and alanine are synthesized (Figure 7).

### Pigments

The partial genomes do not encode any genes for the synthesis of (bacterio)chlorophyll or other proteins required for photosynthesis. The MAGs do, however, encode enzymes for carotenoid biosynthesis, and it is predicted that these organisms should produce cyclic xanthophyll carotenoids. Genes encoding phytoene synthase (*crtB*, 3 copies), phytoene dehydrogenase (*crtI*), lycopene cyclase (*crtY*), β-carotene hydroxylase (*crtZ*), as well as genes possibly encoding pro-ζ-carotene-producing phytoene desaturase (*crtP*) and pro-ζ-carotene desaturase (*crtQ*) were present in the MS MAG. The other two MAGs contained partial gene sets for carotenoid biosynthesis (*crtB*, *crtP* in both; *crtI* only in Chocolate Pots).

### Nitrogen Metabolism

No genes for nitrogen fixation (*nif*) were found in any of the partial genomes, so it is unlikely that these organisms can reduce dinitrogen to ammonia. The presence of glutamine synthetase (GS) as well as glutamate:2-oxoglutarate amidotransferase (GOGAT or glutamate synthase) could suggest that these organisms use ammonia as an N-source. However, similar to *I. album*, no genes for ammonium transporters have yet been found in the partial genomes. Thus, the use of free ammonium as an N-source appears unlikely. More likely, the presence of genes for amino acid and oligopeptide transporters in (almost) all partial genomes suggests that these organisms predominantly use amino acids as nitrogen sources. This is a trait that is also shared with several other mat organisms, including *C. thermophilum*, “*Ca.* T. aerophilum,” and *Synechococcus* spp. (Tank and Bryant, 2015a,b; Tank et al., 2017). As mentioned above, and similar to *M. roseus* (Kadnikov et al., 2013), all three MAGs contain genes indicating an ability to reduce nitrite, but not nitrate (CytS, NosZ, and NrfAH; Figure 7). The absence of nitrate reductase in *M. roseus* was correlated with the observation that nitrate did not stimulate growth, but the organism was able to use nitrite as terminal electron acceptor (Podosokorskaya et al., 2013).

### Transcription of Genes *in situ*

**FIGURE 8:**
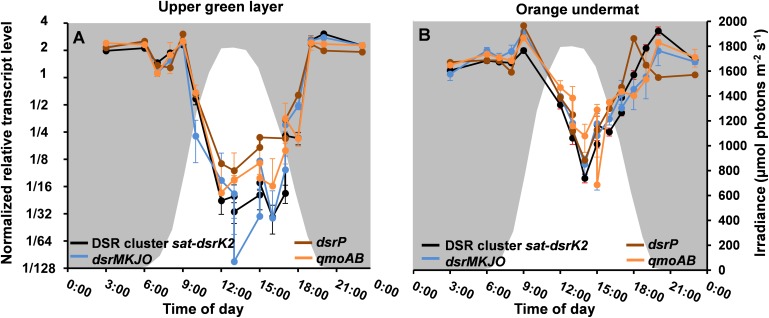
Relative transcript levels (*y*-axis) of DSR-affiliated genes grouped according to the MS contig gene composition shown in Figure 5 across a diel cycle (*x*-axis) in **(A)** MS upper green layer, and **(B)** MS undermat. Black lines represent relative transcript levels of genes *sat-aprAB-qmoC-dsrABCDKLMN*; blue lines represent genes *dsrMKJO*; brown lines represent *dsrP*; orange lines represent *qmoAB.* White areas indicate light periods during the day and irradiance intensities in μmol photons m^-2^ s^-1^ (400–700 nm, secondary *y*-axis), while gray areas indicate dark periods during the night.

Metatranscriptomic data derived from the upper green mat layer of the MS community over a diel cycle were analyzed to identify the relative transcript abundances for all genes assigned to the MAG “MS-B_bin-24,” representing “*Ca.* T. thiotrophicus.” The diel expression patterns for all genes were then subjected to k-means clustering, which revealed six differential expression pattern categories, A–F (Supplementary Figure S2 and Supplementary Table S1). The highest relative transcript levels for most genes occurred at night (clusters D and E) with over 900 genes (ca. 44%) found in these clusters, and many fewer genes had highest transcript levels during the day (cluster C, see below). The relative transcript abundances for the 20 genes associated with sulfate reduction (Table 3) were among those genes with the highest absolute transcript levels and belonged to categories D and E. These genes showed highest relative transcript abundances during the night and transition periods when oxygen levels were low (Figure 8 and Supplementary Table S2). A sharp decrease in transcript levels occurred during the day when cyanobacteria were actively performing photosynthesis and when oxygen levels were maximal. A similar pattern was also observed for “*Ca.* Thermonerobacter” in the mats of Nakabusa; genes associated with DSR showed highest transcript levels during the night and lowest transcript levels during the day (data not shown). Interestingly, in the undermat of MS, at depths where oxygen concentrations never reach the hyperoxic levels of the uppermost part of the mat, transcript levels for the genes associated with sulfur metabolism did not decrease as greatly during the day as in the upper green mat layer (Figure 8, Supplementary Table S2). The transcript levels for a predicted sulfate transporter (MS gene ID 000020_7) matched the expression pattern for the sulfur metabolism genes (Supplementary Table S1). Other respiration-associated genes in the night expression categories (D and E) included genes involved in additional anaerobic processes, such as the reduction of nitrite, nitrous oxide, and thiosulfate (Supplementary Figure S3A and Supplementary Table S2).

Relative transcript levels for the two sets of *nuo* genes were highest at night. Similarly, relative transcript levels for genes encoding ACIII, cytochrome *bd* oxidase, and ATP synthase were also highest at night (Supplementary Figure S3B and Supplementary Table S2). All of these observations support the hypothesis that “*Ca.* T. thiotrophicus” performs anaerobic respiration at night. Interestingly, the relative transcript levels for genes encoding the *aa*_3_-type cytochrome *c* oxidase belong to expression pattern A, which are expressed at highest levels during light–dark transition periods in the evening and the morning (Supplementary Figure S4 and Supplementary Table S2). These transition periods correspond to times when the cyanobacteria are reducing dinitrogen to ammonia, but also represent periods when the oxygen levels in the mat change from oxic to anoxic (evening) and anoxic to oxic (morning).

Transcript levels for genes associated with carbohydrate metabolism, including those for glycolysis/gluconeogenesis and the TCA cycle, as well as those involved in acetate-related metabolism (i.e., acetate kinase and acetyl-CoA acetyltransferase), also belonged to categories D and E and had highest transcript levels at night. However, two genes encoding glycogen synthase belonged to category B and were mostly unchanged throughout the day (Supplementary Figure S5 and Supplementary Tables S1, S2).

Fewer genes (*n* = 222) belonged to the smallest transcript category, C: genes that have highest transcript abundances during the day. Genes involved in oxygen protection, such as superoxide dismutase (MS gene ID 000129_2), peroxiredoxins (000011_73, 000020_4, 000236_9, 000026_30, 000091_19), glutathione peroxidase (000015_37), a nitroreductase (000041_8), and others (Supplementary Table S1), are included in this category (Supplementary Figure S6 and Supplementary Table S2). Other day-genes in category C include the *suf* genes (000129_15-20), which encode proteins involved in iron–sulfur cluster biosynthesis under oxic conditions in many organisms (Supplementary Figure S6 and Supplementary Table S2) (Ayala-Castro et al., 2008) and, various genes coding for molecular chaperones (0054414-6) and several RpoE-like sigma factors (sigma factor 24) (MS gene ID 000006_60, 000002_32, 000100_29, 000020_22, 000036_46) possibly associated with stress conditions (Raina et al., 1995; Supplementary Table S1). The iron chelator hydroxamate (gene ID 004701_1) and a chelator transporter that might presumably be involved in harvesting iron for some of the enzymes mentioned above also showed higher relative transcription levels during the day (Supplementary Table S1, see the section “Discussion”).

Genes encoding flagellar components were largely constitutively expressed, and most belong to diel expression category B with more or less unchanging relative transcription levels throughout the diel cycle. Thus, the transcription patterns did not support the hypothesis that flagella have a specialized function and are only used under specific growth conditions in the mat (Supplementary Table S1). Relative transcript levels for chemotaxis-related genes, however, were higher during the evening and were lower when conditions in the mat become oxic (category D, Supplementary Table S1).

In order to verify that the gene expression patterns observed were accurate representations of the “*Ca.* T. thiotrophicus” ecophysiology and not isolated events, we sampled and analyzed metatranscriptomes at selected times in the 24-h period immediately following the diel cycle examined in this study. These additional time points showed similar transcript levels to those measured in the more complete diel cycle analysis (second day data shown within all metatranscriptome figures; please see time points at 13:00, 15:00, 17:00 for both days data, and time point 14:00 for second day data only. Please note that for some processes, e.g., sulfate reduction, the data on both days are identical and time points overlap). Similarly, the metatranscriptomic analysis from Nakabusa included independent samples at time points on two different days as a control for day to day reproducibility. In the samples from Nakabusa, similar transcript levels were also found for samples taken at the same time points on consecutive days (data not shown).

## Discussion

### “*Ca.* Thermonerobacter thiotrophicus”: A Member of the *Chlorobi*-Lineage 5 (OPB56, “*Ca.* Kapabacteria”) With Dissimilatory Sulfur Metabolism

In this study we describe the initial genomic and transcriptomic characterization of “*Ca.* T. thiotrophicus,” a member of the *Bacteroidetes*/*Chlorobi* group from MS microbial mats with dissimilatory sulfur metabolism, and predicted sulfate-reducing directionality. “*Ca.* T. thiotrophicus” is the first described putatively SRM from the *Bacteroidetes*/*Chlorobi* group, among which to this point only (phototrophic) members with sulfide-oxidizing, dissimilatory sulfur metabolism have been observed. SRM were first shown to occur in the microbial mats associated with MS on the basis of *dsrAB* cloning experiments, microelectrode measurements, and enzymatic assays (Dillon et al., 2007). Sequences from this earlier study, labeled as “unidentified clade-2,” clustered phylogenetically with the “environmental supercluster” of reductive DsrAB sequences. These sequences were also detected in this metagenomic study, and a MAG for the uncultured SRM was obtained. Additional MAGs containing highly similar *dsrAB* gene sequences to those in MS were identified in metagenomes from Nakabusa hot springs in Japan and the iron-rich, Chocolate Pots hot spring in YNP.

No cultured representative of the “environmental supercluster” of *dsrAB* sequences has yet been described, and no taxonomic classification of the *dsrAB*-containing organisms in MS had been possible until this study. Recently, (meta)genomics have allowed insights into the functional potentials of many uncultured organisms, and partial genomes of uncultured members of different phyla, including *Acidobacteria*, *Nitrospira*, *Chloroflexi*, “*Ignavibacteria*,” and a number of “*Candidatus*” phyla have been affiliated with this largely uncharacterized “environmental supercluster” (Anantharaman et al., 2018; Hausmann et al., 2018; Zecchin et al., 2018). Using ribosomal genes present in the Nakabusa MAG as well as other phylogenetic marker genes from the three MAGs studied, we were able to identify the uncultured, putative SRM from these hot springs as members of the “*Chlorobi* lineage 5,” also known as “clade OPB56,” or “*Ca.* Kapabacteria” (Iino et al., 2010; Kantor et al., 2015). Previous partial genomes of representatives of this clade suggested that these organisms are chemoheterotrophs that metabolize small organic molecules and are broadly distributed in thermal environments (Hiras et al., 2016). However, DSR was not indicated by those studies. We propose the name of “*Candidatus* Thermonerobacter” for this hot spring-associated SRM from the OPB56 clade, with the member from MS as “*Candidatus* Thermonerobacter thiotrophicus.”

Moreover, we propose that the *dsrAB*-containing “*Ignavibacteria*” genome of MAG GWB2_35_12 (GenBank WGS entry MGZV) identified by Anantharaman et al. (2018) should be re-classified as an OPB56 member, a sister clade to *Ignavibacteraceae*. Based on phylogenetic analyses of the 16S rRNA (Figure 4) and phylogenetic marker genes (Figure 3), this organism is very similar and shares most of the genomic properties with the organisms described in this study, although it comes from a very different environment, the groundwater aquifer system at Rifle, CO, United States. Some minor differences in the organization of the genes for sulfate reduction are observed (Figure 5), but overall this organism appears to be quite similar to those described here from hot springs.

### Metabolic Prediction and Versatility/Activity *in situ*

Despite the low sulfate concentrations in the mat (<200 μM), substantial rates of sulfate reduction were measured in a previous study of MS (Dillon et al., 2007). The overall activity was attributed to *Thermodesulfovibrio* sp. as well as to organisms associated with three unidentified *dsrAB* clone sequences retrieved from the mats. In addition, although there is almost no measurable sulfide in the spring water (0.003 mg/l = 0.08 μM, USGS report 2001–2002, McCleskey et al., 2004), the MS mats contain significant numbers of sulfide-oxidizing chlorophototrophs (e.g., *Chloroflexus* sp., *Roseiflexus* sp., “*Ca*. Chloranaerofilum corporosum,” and possibly even *Synechococcus* spp.) (Thiel et al., 2016, 2017; Tank et al., 2017), as well as sulfur-oxidizing members, such as *Thermocrinis* sp. (Thiel et al., 2017), which would provide sulfate for DSR in the mats. Active sulfate reduction during evening and night hours is further supported by the observation that sulfide accumulates in the mat during the night but is undetectable during the day (E. Trampe, M. Lichtenberg, V. Thiel, M. Kühl, unpublished data; Dillon et al., 2007).

Given the predicted significance of a tightly coupled sulfur cycle, identifying organisms responsible for sulfate reduction had been an important goal of ongoing studies on the MS mats and those associated with similar hot springs. Our data indicate that “*Ca.* T. thiotrophicus” is involved in such sulfur cycling. Relative transcript levels for the *dsr* genes in this organism varied from night to day to a far greater extent than any other transcripts in these organisms, including those associated with other anaerobic processes such as nitrite and thiosulfate respirations. The patterns of night/day expression variation were consistent in the upper layers and undermats of both MS and Nakabusa hot spring. Interestingly, in the undermat of MS, at depths where oxygen concentrations never reach the hyperoxic levels of the uppermost part of the mat, transcripts levels for the genes associated with sulfur metabolism did not decrease as greatly during the day as they did in the upper green mat layer (Figure 7). This possibly indicates that these genes are regulated by environmental oxygen conditions and that active expression of the genes for sulfate reduction occurs at times when the mats are anoxic. In fact, most genes involved in energy conservation, including those involved in carbohydrate oxidation, electron transport and ATP synthase exhibited higher relative transcript levels at night than during the day.

In contrast to the large number of genes and processes with a nighttime transcription pattern, we found few genes that could be involved in energy conservation with higher relative transcript levels during the day. These include a putative 2-oxovalerate:ferredoxin oxidoreductase (VOR), an aldo/keto oxidoreductase with homology to oxidoreductases associated with the oxidation of amino acids, two genes coding for ferredoxin, and two genes with homology to cytochrome oxidase biosynthesis components (Supplementary Figure S7 and Supplementary Table S2). Given the lack of additional metabolic transcripts during the day, it is unclear whether the products of these genes participate in energy conservation, and if so, whether they contribute to a common metabolic process or act independently of one another. In contrast to the relatively small number of metabolic genes with highest transcript levels during the day, genes with products involved in stress responses, such as those associated with oxygen protection, and *suf* genes used to produce FeS clusters under oxygen-stress conditions (Jang and Imlay, 2010; Zang et al., 2017; Supplementary Figure S6 and Supplementary Table S2) are highly represented in the day-time expression cluster. These patterns reflect the significant oxygen levels this organism sees during the day. Lastly, the partial genomes encode two oxygen-dependent terminal oxidases (*caa*_3_ and *bd*-type), of which cytochrome oxidase *caa*_3_ shows a dual transcription peak pattern during the day, peaking in the morning and a second time in the evening (Supplementary Figure S5 and Supplementary Table S2). This might point toward active oxygen respiration in this organism, possibly during times of low oxygen concentrations. Although no previously isolated natural SRM has been demonstrated to grow by aerobic respiration, a new study has shown that this ability can evolve under oxygen-driven conditions (Schoeffler et al., 2018). This finding supports the idea that novel and uncultured hot spring organisms may also have this capability. As *Desulfovibrio* species contain the electron-transport complexes required for both sulfate and oxygen reduction (Figure 7), only a few mutations, presumably involving either inactivation or overexpression of the gene encoding heterodisulfide reductase, were required to permit the emergence of oxygen-respiration enabled growth (Schoeffler et al., 2018). Similarly, genes for both systems, sulfate and oxygen respiration, were detected in the “*Ca.* T. thiotrophicus” MAG under strongly aerobic day-time conditions, which would favor the evolution of a flexible, alternating facultative anaerobic metabolism. However, the observation that most genes, including those involved in electron transfer, are transcribed during the night might argue against energy production by aerobic respiration, at least under highly oxic conditions. Alternatively, this cytochrome oxidase might function in oxygen protection, as has been suggested to occur in some strictly anaerobic GSB (Li et al., 2009).

Sequences affiliated with “*Ca*. Thermonerobacter thiotrophicus,” the putative, sulfate-reducing member of OPB56 (*Bacteroidetes*/*Chlorobi*), have been detected in both the upper layer and undermat of MS. Based on amplicon data for MS-B_OTU-29 (Figure 4; Thiel et al., 2016) relative abundance of this member does not differ between the two layers. The upper layer, in particular, experiences a strong diel cycling of oxygen concentrations that can reach up to 800% air saturation. The presence and transcription of oxygen protection genes in the *Bacteroidetes*/*Chlorobi* member during the day support this hypothesis; most genes with high relative transcript levels during the day belong to the categories of oxygen and stress protection. Based on these data we predict that pathways of conserving energy in “*Ca.* T. thiotrophicus” are active during the anoxic periods of the diel cycle whereas, during the oxic periods, cells respond to oxygen stress by activating mechanisms that slow down or pause its metabolism. As expected from these predictions, shifts in relative transcript levels are not as pronounced in the undermat, where the oxygen levels do not reach the supersaturated levels in the upper green layer. However, an alternating aerobic/anaerobic metabolism involving sulfate as well as oxygen respiration cannot be excluded but can only be experimentally verified in future studies when pure cultures of these novel organisms are available.

### SRM or SOM?

In natural systems, the oxidation state of sulfur can range from -2 to +6 and can support microbial energy metabolism as a reductant or oxidant. DSR is performed by microorganisms using sulfate as a terminal electron acceptor during anaerobic respiration (“sulfate respiration”), whereas in dissimilatory sulfur oxidation, sulfur compounds are used as electron donors in respiratory or photoautotrophic metabolism. Despite the different directionality of the reactions, a similar set of DSR genes is found in both SRM and SOM, which indicates a common origin from an early ancestor (Wagner et al., 1998; Grein et al., 2013; Müller et al., 2015). Although DSR genes have been found in both SRM and SOM, specific patterns and differences occur in the two groups.

The *dsr* genes encoding the Hrd-like menaquinol-oxidizing enzyme (*dsrTMKJOP*) as well as the genes *sat-aprBA-qmoABC* are not unique to SRM, but rather are also present in many SOM. Thus, the presence of these genes is not diagnostic for either SRM or SOM. The *dsrAB* genes, on the other hand, have long been used as phylogenetic markers to distinguish between these two metabolic modes, and to a more limited extent to identify known phylogenetic groups. Based on DsrAB phylogeny, the uncultured organisms described here were affiliated with “unclassified environmental sequences” within the “environmental supercluster 1” of unidentified and uncultured SRM (Müller et al., 2015). This affiliation is strongly suggestive that the hot-spring organisms perform DSR. Further support for this contention is found by the absence of the *dsrEFH* genes as well as *aprM*, which are usually present in SOM but not SRM. However, the recent findings of Thorup et al. (2017), who describe a novel deltaproteobacterium, *D. alkaliphilus*, which contains all genes phylogenetically characteristic of a SRM, but performs sulfide oxidation as its sole mode of dissimilatory sulfur metabolism, challenges the above-mentioned arguments. Nevertheless, the presence of all SRM “marker genes” as well as the absence of all SOM “marker genes,” in combination with the very high relative transcript levels for all genes for sulfate reduction during the anoxic night period, lead us to predict that these uncultured organisms perform sulfate reduction during periods when their mat environments are anoxic. Similarly, the relatively low transcript levels during oxic day times, as well as non-detectable sulfide concentrations in the microbial mats during the day (van der Meer et al., 2005; E. Trampe, M. Lichtenberg, V. Thiel, and M. Kuehl, unpublished data) do not support a metabolism based on sulfide oxidation coupled to aerobic respiration, as has been discussed for the *dsrAB*-carrying *Acidobacteria*, which carry a similar genome organization of the DSR pathway as observed here (Hausmann et al., 2018). Finally, the third alternative, using the DSR-related genes for anaerobic sulfide-oxidation coupled with dissimilatory nitrate reduction to ammonium (DNRA) as reported for *D. alkaliphilus* (Thorup et al., 2017), is considered to be unlikely because these organsims lack a nitrate reductase as well as any complete pathway for carbon fixation.

### Implications for Our Understanding of the Evolution of Dissimilatory Sulfur Metabolism

For about a century, sulfur-oxidizing GSB were the only known members of the phylum *Chlorobi*, and the two terms were often used synonymously. The detection of DSR genes in putative sulfate-reducing chemoheterotrophic organisms within the same (super)phylum presents new and unexpected evidence in the evolutionary history of dissimilatory sulfur metabolism. Culture-independent studies, principally 16S rRNA gene analyses, have indicated that a far greater diversity exists within this phylum and have disclosed a substantial variety of uncultivated members affiliated within this group. However, based on the limited metabolic diversity of GSB, initially these distant relatives were incorrectly assumed to be sulfide-oxidizing, anaerobic chlorophototrophs (Iino et al., 2010; Liu et al., 2012a). The isolation and characterization of two chemoorganoheterotrophic strains, *I. album* (Iino et al., 2010; Liu et al., 2012a) and *M. roseus* (Kadnikov et al., 2013; Podosokorskaya et al., 2013), clearly demonstrated that the *Chlorobi*/*Ignavibacteria*-like (super)phylum encompassed much greater metabolic diversity than previously imagined. Presently, this group comprises five lineages in addition to *Chlorobia*. The two non-phototrophic isolates mentioned above are affiliated with “*Chlorobi* lineage 1,” which was proposed as a new class, *Ignavibacteria* (Iino et al., 2010), but it has also been suggested to represent a novel phylum, *Ignavibacteriae* (Podosokorskaya et al., 2013). No isolated representatives have yet been described for any of the other four lineages, but partial genomes available for members of “*Chlorobi* lineage 5” (also known as OPB56 group; recently suggested to represent a novel phylum, “*Ca.* Kapabacteria;” Kantor et al., 2015) indicate that this lineage includes organisms predicted to be facultative anaerobic chemoheterotrophs, possibly with a putatively predatory lifestyle (Kantor et al., 2015; Hiras et al., 2016). However, no sulfate-reducing or chemotrophic sulfur-oxidizing member of the *Chlorobi*/*Ignavibacteria-*like superphylum has yet been isolated and characterized.

As discussed above, GSB (and other SOM) contain a set of DSR genes that they utilize for sulfide/sulfur oxidation, but that are very similar to those of sulfate-reducing organisms. Interestingly, not all chlorophototrophic members of the *Chlorobi* contain DSR genes. *Chloroherpeton thalassium*, an early diverging chlorophototrophic member of the *Chlorobiales*, lacks the rDSR system (Frigaard and Bryant, 2008) and only possesses sulfide:quinone reductase, like sulfide-oxidizing members of the *Chloroflexi*. Furthermore, the iron-oxidizing anaerobes, *Chlorobium ferrooxidans* and *Chlorobium phaeoferrooxidans* and the photoheterotrophic aerobe, “*Ca*. T. aerophilum,” lack all of the genes for sulfide oxidation to sulfite. This and additional phylogenetic analyses suggest that the DSR pathway may have been acquired by the *Chlorobi* lineage after the divergence of *Chloroherpeton*-like organisms (Frigaard and Bryant, 2008; Liu et al., 2012b). On the other hand, DSR genes in sulfur-oxidizing, chlorophototrophic GSB, seem to represent a hybrid stage or an evolutionary chimera, in which some characteristics clearly are associated with SOM (such as their *dsrAB* phylogenetic affiliation), while other characteristics and genes are shared with SRM (such as *dsrTMKJOP*). This chimeric status of the DSR system in the sulfur-oxidizing, phototrophic *Chlorobi* suggests that this group did not inherit or acquire its genes from a typical SRM. In terms of the evolution of the DSR in the *Chlorobi*, three hypothetical scenarios can be considered: (1) the DSR genes were first acquired in one of the groups and were subsequently transferred horizontally to the other *Chlorobi*, thereby changing the utilization of the genes from oxidation to reduction or vice versa; (2) the DSR genes were acquired at an evolutionary earlier stage by a putative SRM, then loss of the genes occurred in some lineages and the genes were converted from reductive to oxidative dissimilatory metabolism in another lineage; or (3) a horizontal acquisition of rDSR genes in GSB after the loss of the sulfate-reducing set of DSR genes after the divergence from the chemotrophic *Chlorobi* lineage(s). It has been shown that GSB are rather susceptible to HGT, which could further support the hypothesis of a possible acquisition of DSR-related genes from two different sources (Llorens-Marès et al., 2017). It should further be noted that chlorophototrophy in the *Chlorobi* was probably obtained by HGT, and if this was the case in an organism that contained genes for DSR, there would have been strong selection pressure to alter the directionality of the reactions of sulfur metabolism. A detailed phylogenetic analysis of DSR-related genes will be performed to address these questions in the future.

### Concluding Comments

In this study partial genomes assembled from metagenomic data are presented, which describe putative, novel SRM, tentatively named “*Ca.* Thermonerobacter (thiotrophicus)” that occur in hot spring mat communities. The three MAGs described here contain complete (or nearly complete) sets of genes encoding all known enzymes required for DSR. Active sulfate reduction has previously been reported to occur in one of the three hot-spring microbial mats. Metatranscriptomic studies performed on samples collected over a complete diel cycle for two of the hot springs showed that relative transcript levels for all 20 genes related to DSR were highest during the night. We hypothesize that the novel but presently uncultured organisms perform DSR at night. The presence of oxidative terminal cytochrome c oxidases in the genomes, and their transcription during the day (Supplementary Figures S5, S7C, Supplementary Table S2) might further indicate the facultative ability to also perform aerobic respiration. Future studies *in situ* and/or with enrichment or axenic cultures of these novel DSR-containing organisms will test this hypothesis and should conclusively demonstrate whether these “*Chlorobi* lineage 5” (OPB56, “*Ca.* Kapabacteria”) members, “*Ca.* Thermonerobacter (thiotrophicus),” indeed are SRM or SOM.

## Author Contributions

VT conducted sampling, DNA and RNA extraction, metagenome binning for MS samples (YNP, WY, United States), as well as data analysis of MAGs. AGC conducted RNAseq data analysis for MS (YNP, WY, United States) and NK (Japan) samples and MAGs. NF conducted sampling, DNA extraction, and metagenome binning for CP samples (YNP, WY, United States). JM conducted sampling, DNA and RNA extraction, and binning for NK samples (Japan). MT conducted sampling for MS (YNP, WY, United States) and NK (Japan) samples, contributed to DNA and RNA extraction, data analysis and interpretation of MAGs. ER and EB planned and funded the study at CP (YNP, WY, United States). DW and DB planned and funded the study at MS (YNP, WY, United States). VT and SH planned and funded the study at NK (Japan). VT, AGC, and DB wrote the manuscript. All co-authors contributed to the manuscript with sections of their expertise, feedback, and fruitful discussions.

## Conflict of Interest Statement

The authors declare that the research was conducted in the absence of any commercial or financial relationships that could be construed as a potential conflict of interest.

## Footnotes

1 https://www.biotech.wisc.edu

2 http://www.clcbio.com

3 http://www.bioinformatics.babraham.ac.uk/projects/fastqc/

4 https://www.bioconductor.org/

5 https://img.jgi.doe.gov/cgi-bin/mer/main.cgi

6 https://www.ncbi.nlm.nih.gov/assembly

7 http://www.ggvaidya.com/taxondna/

8 https://www.arb-silva.de/

9 https://www.arb-silva.de/download/arb-files/

10 https://jgi.doe.gov/data-and-tools/

11 http://enve-omics.ce.gatech.edu/ani/

12 https://img.jgi.doe.gov/

## Supplementary Material

Figure S1 Phylogenetic tree based on DsrAB amino acid sequences displaying the phylogenetic affiliation of the putative thermophilic SRM *Bacteroidetes–Chlorobi* group member (red box within the Environmental Supercluster in the reductive-type DsrAB clade). Sequences of GSB, SOM members of the *Chlorobi* cluster with the rDSR (sulfur oxidizing) DsrAB clade. The tree was calculated from with the publicly available DsrAB ARB database (Müller et al., 2015; http://www.microbial-ecology.net/download). New sequences were added using the parsimony method without changing the tree topology.

Figure S2 Heatmaps showing expression patterns of “*Ca.* T. thiotrophicus” genes throughout a diel cycle in the upper green layer of MS (sampling times shown at the bottom). Red = high relative transcript abundance; green = low relative transcript abundance; black = no transcripts detected. Higher color intensity reflects relatively higher transcript levels. Genes are clustered according to their major patterns of expression as described in the text. Red **(A)** Genes (390) with expression peaks at 09:00 and 18:00. **(B)** Genes (546) with variable expression throughout the day. **(C)** Genes (222) with strong daytime expression. **(D)** Genes (706) with weak nighttime expression. **(E)** Genes (248) with strong nighttime expression. **(F)** Genes (52) with maximal transcript levels at 18:00.

Figure S3 Relative transcript levels (*y*-axis) across a diel cycle (*x*-axis) for in the upper green layer of MS of genes associated with anaerobic respiration **(A)** and other electron transfer processes **(B)**. White areas indicate light periods during the day and irradiance intensities in μmol photons m^-2^ s^-1^ (400–700 nm, secondary *y*-axis), while gray areas indicate dark periods during the night.

Figure S4 Relative transcript levels (*x*-axis) of *aa*_3_-type cytochrome *c* oxidase across a diel cycle (*x*-axis) in the upper green mat layer of MS. White areas indicate light periods during the day and irradiance intensities in μmol photons m^-2^ s^-1^ (400–700 nm, secondary *y*-axis), while gray areas indicate dark periods during the night.

Figure S5 Relative transcription levels (*y*-axis) of genes involved in glycolysis, the TCA cycle, and other central carbon metabolism pathways across a diel cycle (*x*-axis) in MS MAG (MS-B_bin-24), in the upper green mat layer of MS. White areas indicate light periods during the day and irradiance intensities in μmol photons m^-2^ s^-1^ (400–700 nm, secondary *y*-axis), while gray areas indicate dark periods during the night.

Figure S6 Relative transcript levels (*y*-axis) of genes associated with oxygen stress/oxic conditions during a diel cycle (*y*-axis) in the upper green mat layer of MS. White areas indicate light periods during the day and irradiance intensities in μmol photons m^-2^ s^-1^ (400–700 nm, secondary *y*-axis), while gray areas indicate dark periods during the night.

Figure S7 Relative transcript levels (*y*-axis) of selected day genes across a diel cycle (*x*-axis) in the upper green mat layer of MS. **(A)** Putative 2-oxovalerate:ferredoxin oxidoreductase (VOR) and aldo/keto oxidoreductase; **(B)** ferredoxins; **(C)** cytochrome biosynthesis genes. White areas indicate light periods during the day and irradiance intensities in μmol photons m^-2^ s^-1^ (400–700 nm, secondary *y*-axis), while gray areas indicate dark periods during the night.

Table S1 Excel file with information of the genes included in each of the expression categories, A–F.

Table S2 Excel file with information of the genes included in the expression figures.

## References

[B1] Aiba H. Adhya S. de Crombrugghe B. (1981). Evidence for two functional *gal* promoters in intact *Escherichia coli* cells. *J. Biol. Chem.* 256 11905–11910. 10.1159/000335853 6271763

[B2] Alneberg J. Bjarnason B. S. R. De Bruijn I. Schirmer M. Quick J. Ijaz U. Z. (2014). Binning metagenomic contigs by coverage and composition. *Nat. Methods* 11 1144–1146. 10.1038/nmeth.3103 25218180

[B3] Altschul S. F. Gish W. Miller W. Myers E. W. Lipman D. J. (1990). Basic local alignment search tool. *J. Mol. Biol.* 215 403–410. 10.1016/S0022-2836(05)80360-2 2231712

[B4] Anantharaman K. Brown C. T. Hug L. A. Sharon I. Castelle C. J. Probst A. J. (2016). Thousands of microbial genomes shed light on interconnected biogeochemical processes in an aquifer system. *Nat. Commun.* 7:13219. 10.1038/ncomms13219 27774985 PMC5079060

[B5] Anantharaman K. Hausmann B. Jungbluth S. P. Kantor R. S. Lavy A. Warren L. A. (2018). Expanded diversity of microbial groups that shape the dissimilatory sulfur cycle. *ISME J.* 12 1715–1728. 10.1038/s41396-018-0078-0 29467397 PMC6018805

[B6] Ayala-Castro C. Saini A. Outten F. W. (2008). Fe-S cluster assembly pathways in bacteria. *Microbiol. Mol. Biol. Rev.* 72 110–125. 10.1128/MMBR.00034-07 18322036 PMC2268281

[B7] Aziz R. K. Bartels D. Best A. A. DeJongh M. Disz T. Edwards R. A. (2008). The RAST Server: rapid annotations using subsystems technology. *BMC Genom.* 9:75. 10.1186/1471-2164-9-75 18261238 PMC2265698

[B8] Ball J. W. McCleskey R. B. Nordstrom D. K. Holloway J. Verplanck P. L. (2004). *Water-Chemistry Data for Selected Springs, Geysers and Streams in Yellowstone National Park, Wyoming, 2001-2002.* Reston, VA: U. S. Geological Survey.

[B9] Bowles M. W. Mogollón J. M. Kasten S. (2014). Global rates of marine sulfate reduction and implications for sub–sea-floor metabolic activities. *Science* 344 889–891. 10.1038/35351 24812207

[B10] Brock T. D. (1967). Micro-organisms adapted to high temperatures. *Nature* 214 882–885. 10.1038/214882a0 6054968

[B11] Bryant D. A. Garcia Costas A. M. Maresca J. A. Chew A. G. M. Klatt C. G. Bateson M. M. (2007). Candidatus *Chloracidobacterium thermophilum*: an aerobic phototrophic Acidobacterium. *Science* 317 523–526. 10.1126/science.1143236 17656724

[B12] Bryant D. A. Liu Z. Li T. Zhao F. Costas A. M. G. Klatt C. G. (2012). “Comparative and functional genomics of anoxygenic green bacteria from the taxa *Chlorobi*, *Chloroflexi*, and *Acidobacteria*,” in *Functional Genomics and Evolution of Photosynthetic Systems. Advances in Photosynthesis and Respiration* Vol. 33 eds Burnap R. L. Vermaas W. F. J. (Basingstoke: Springer Nature), 47–102. 10.1007/978-94-007-1533-2

[B13] Buckel W. Thauer R. K. (2018a). Flavin-based electron bifurcation, ferredoxin, flavodoxin, and anaerobic respiration with protons (Ech) or NAD+ (Rnf) as electron acceptors: a historical review. *Front. Microbiol.* 9:401. 10.3389/fmicb.2018.00401 29593673 PMC5861303

[B14] Buckel W. Thauer R. K. (2018b). Flavin-based electron bifurcation, a new mechanism of biological energy coupling. *Chem. Rev.* 118 3862–3886. 10.1021/acs.chemrev.7b00707 29561602

[B15] Canfield D. E. Stewart F. J. Thamdrup B. De Brabandere L. Dalsgaard T. Delong E. F. (2010). A cryptic sulfur cycle in oxygen minimum zone waters off the Chilean coast. *Science* 330 1375–1378. 10.1126/science.1196889 21071631

[B16] Dahl C. (2017). “Sulfur metabolism in phototrophic bacteria,” in *Modern Topics in the Phototrophic Prokaryotes - Metabolism, Bioenergetics, and Omics*, ed. Hallenbeck P. (Berlin: Springer International Publishing), 27–66. 10.1007/978-3-319-51365-2

[B17] Darling A. E. Jospin G. Lowe E. Matsen F. A. IV Bik H. M. Eisen J. A. (2014). PhyloSift: phylogenetic analysis of genomes and metagenomes. *PeerJ* 2:e243. 10.7717/peerj.243 24482762 PMC3897386

[B18] Dhillon A. Teske A. Dillon J. Stahl D. A. Sogin M. L. (2003). Molecular characterization of sulfate-reducing bacteria in the Guaymas Basin. *Appl. Environ. Microbiol.* 69:2765 10.1128/AEM.69.5.2765 PMC154542 12732547

[B19] Dick G. J. Andersson A. F. Baker B. J. Simmons S. L. Thomas B. C. Yelton A. P. (2009). Community-wide analysis of microbial genome sequence signatures. *Genome Biol.* 10:R85. 10.1186/gb-2009-10-8-r85 19698104 PMC2745766

[B20] Dillon J. G. Fishbain S. Miller S. R. Bebout B. M. Habicht K. S. Webb S. M. (2007). High rates of sulfate reduction in a low-sulfate hot spring microbial mat are driven by a low level of diversity of sulfate-respiring microorganisms. *Appl. Environ. Microbiol.* 73 5218–5226. 10.1128/AEM.00357-07 17575000 PMC1950965

[B21] Eisen M. B. Spellman P. T. Brown P. O. Botstein D. (1998). Cluster analysis and display of genome-wide expression patterns. *Proc. Natl. Acad. Sci. U.S.A.* 95 14863–14868. 10.1073/pnas.95.25.14863 9843981 PMC24541

[B22] Forte E. Borisov V. B. Vicente J. B. Giuffrè A. (2017). Cytochrome *bd* and gaseous ligands in bacterial physiology. *Adv. Microb. Physiol.* 71 171–234. 10.1016/bs.ampbs.2017.05.002 28760322

[B23] Fortney N. W. He S. Converse B. J. Beard B. L. Johnson C. M. Boyd E. S. (2016). Microbial Fe(III) oxide reduction potential in Chocolate Pots hot spring, Yellowstone National Park. *Geobiology* 14 255–275. 10.1111/gbi.12173 26750514

[B24] Fortney N. W. He S. Kulkarni A. Friedrich M. W. Holz C. Boyd E. S. (2018). Stable isotope probing of microbial iron reduction in Chocolate Pots hot spring, Yellowstone National Park. *Appl. Environ. Microbiol.* 84:e02894–17. 10.1128/AEM.02894-17 29602784 PMC5960972

[B25] Frigaard N. U. Bryant D. A. (2008). “Genomic Insights into the sulfur metabolism of phototrophic green sulfur bacteria,” in *Sulfur Metabolism in Phototrophic Organisms. Advances in Photosynthesis and Respiration* Vol. 27 eds Hell R. Dahl C. Knaff D. Leustek T. (Dordrecht: Springer), 337–355.

[B26] Frigaard N. U. Dahl C. (2008). Sulfur metabolism in phototrophic sulfur bacteria. *Adv. Microb. Physiol.* 54 103–200. 10.1016/S0065-2911(08)00002-7 18929068

[B27] Fuchs G. (2011). Alternative pathways of carbon dioxide fixation: insights into the early evolution of life? *Annu. Rev. MIcrobiol.* 65 631–658. 10.1146/annurev-micro-090110-102801 21740227

[B28] González J. M. Pinhassi J. Fernández-Gómez B. Coll-Lladó M. González-Velázquez M. Puigbò P. (2011). Genomics of the proteorhodopsin-containing marine flavobacterium *Dokdonia* sp. strain MED134. *Appl. Environ. Microbiol.* 77 8676–8686. 10.1128/AEM.06152-11 22003006 PMC3233072

[B29] Goris J. Konstantinidis K. T. Klappenbach J. A. Coenye T. Vandamme P. Tiedje J. M. (2007). DNA-DNA hybridization values and their relationship to whole-genome sequence similarities. *Int. J. Syst. Evol. Microbiol.* 57 81–91. 10.1099/ijs.0.64483-0 17220447

[B30] Grein F. Ramos A. R. Venceslau S. S. Pereira I. A. C. (2013). Unifying concepts in anaerobic respiration: insights from dissimilatory sulfur metabolism. *Biochim. Biophys. Acta Bioenerg.* 1827 145–160. 10.1016/j.bbabio.2012.09.001 22982583

[B31] Hausmann B. Pelikan C. Herbold C. W. Köstlbacher S. Albertsen M. Eichorst S. A. (2018). Peatland *Acidobacteria* with a dissimilatory sulfur metabolism. *ISME J.* 12 1729–1742. 10.1038/s41396-018-0077-1 29476143 PMC6018796

[B32] Hinsley A. P. Berks B. C. (2002). Specificity of respiratory pathways involved in the reduction of sulfur compounds by Salmonella enterica. *Microbiology* 148 3631–3638. 10.1099/00221287-148-11-3631 12427953

[B33] Hiras J. Wu Y.-W. Eichorst S. A. Simmons B. A. Singer S. W. (2016). Refining the phylum *Chlorobi* by resolving the phylogeny and metabolic potential of the representative of a deeply branching, uncultivated lineage. *ISME J.* 10 833–845. 10.1038/ismej.2015.158 26325358 PMC4796922

[B34] Hittel D. S. Voordouw G. (2000). Overexpression, purification and immunodetection of DsrD from *Desulfovibrio vulgaris* Hildenborough. *Antonie Van Leeuwenhoek* 77 271–280. 10.1023/A:1002449227469 15188893

[B35] Holkenbrink C. Barbas S. O. Mellerup A. Otaki H. Frigaard N. U. (2011). Sulfur globule oxidation in green sulfur bacteria is dependent on the dissimilatory sulfite reductase system. *Microbiology* 157 1229–1239. 10.1099/mic.0.044669-0 21233162

[B36] Iino T. Mori K. Uchino Y. Nakagawa T. Harayama S. Suzuki K. I. (2010). *Ignavibacterium album* gen. nov., sp. nov., a moderately thermophilic anaerobic bacterium isolated from microbial mats at a terrestrial hot spring and proposal of *Ignavibacteria* classis nov., for a novel lineage at the periphery of green sulfur bacteria. *Int. J. Syst. Evol. Microbiol.* 60 1376–1383. 10.1099/ijs.0.012484-0 19671715

[B37] Jang S. Imlay J. A. (2010). Hydrogen peroxide inactivates the *Escherichia coli* Isc iron-sulfur assembly system, and OxyR induces the Suf system to compensate. *Mol. Microbiol.* 78 1448–1467. 10.1111/j.1365-2958.2010.07418.x 21143317 PMC3051806

[B38] Jørgensen B. B. (1977). The sulfur cycle of a coastal marine sediment (Limfjorden, Denmark). *Limnol. Oceanogr.* 22 814–832. 10.4319/lo.1977.22.5.0814

[B39] Jørgensen B. B. (1982). Mineralization of organic matter in the sea bed - The role of sulphate reduction. *Nature* 296 643–645. 10.1038/296643a0

[B40] Jørgensen B. B. Fenchel T. (1974). The sulfur cycle of a marine sediment model system. *Mar. Biol.* 24 189–201. 10.1007/BF00391893

[B41] Kadnikov V. V. Mardanov A. V. Podosokorskaya O. A. Gavrilov S. N. Kublanov I. V. Beletsky A. V. (2013). Genomic analysis of *Melioribacter roseus*, facultatively anaerobic organotrophic bacterium representing a novel deep lineage within *Bacteriodetes/Chlorobi* group. *PLoS One* 8:e53047. 10.1371/journal.pone.0053047 23301019 PMC3534657

[B42] Kantor R. S. van Zyl A. W. van Hille R. P. Thomas B. C. Harrison S. T. L. Banfield J. F. (2015). Bioreactor microbial ecosystems for thiocyanate and cyanide degradation unravelled with genome-resolved metagenomics. *Environ. Microbiol.* 17 4929–4941. 10.1111/1462-2920.12936 26031303

[B43] Kato S. Sakai S. Hirai M. Tasumi E. Nishizawa M. Suzuki K. (2018). Long-term cultivation and metagenomics reveal ecophysiology of previously uncultivated thermophiles involved in biogeochemical nitrogen cycle. *Microbes. Environ.* 33 107–110. 10.1264/jsme2.ME17165 29459499 PMC5877337

[B44] Kerepesi C. Bánky D. Grolmusz V. (2014). AmphoraNet: the webserver implementation of the AMPHORA2 metagenomic workflow suite. *Gene* 533 538–540. 10.1016/j.gene.2013.10.015 24144838

[B45] Klatt C. G. Liu Z. Ludwig M. Kühl M. Jensen S. I. Bryant D. A. (2013). Temporal metatranscriptomic patterning in phototrophic *Chloroflexi* inhabiting a microbial mat in a geothermal spring. *ISME J.* 7 1775–1789. 10.1038/ismej.2013.52 23575369 PMC3749495

[B46] Klatt C. G. Wood J. M. Rusch D. B. Bateson M. M. Hamamura N. Heidelberg J. F. (2011). Community ecology of hot spring cyanobacterial mats: predominant populations and their functional potential. *ISME J.* 5 1262–1278. 10.1038/ismej.2011.73 21697961 PMC3146275

[B47] Klein M. Friedrich M. Roger A. J. Fishbain S. Abicht H. Blackall L. L. (2001). Multiple lateral transfers of dissimilatory sulfite reductase genes between major lineages of sulfate-reducing prokaryotes. *J. Bacteriol.* 183 6028–6035. 10.1128/JB.183.20.6028 11567003 PMC99682

[B48] Kumar S. Stecher G. Tamura K. (2016). MEGA7: molecular evolutionary genetics analysis version 7.0 for bigger datasets. *Mol. Biol. Evol.* 33 1870–1874. 10.1093/molbev/msw054 27004904 PMC8210823

[B49] Langmead B. Salzberg S. (2012). Fast gapped-read alignment with Bowtie 2. *Nat. Methods* 9 357–359. 10.1038/nmeth.1923 22388286 PMC3322381

[B50] Larsson A. (2014). AliView: a fast and lightweight alignment viewer and editor for large datasets. *Bioinformatics* 30 3276–3278. 10.1093/bioinformatics/btu531 25095880 PMC4221126

[B51] Leloup J. Quillet L. Berthe T. Petit F. (2006). Diversity of the *dsrAB* (dissmilatory sulfite reductase) gene sequences retrieved from two contrasting mudflats of the Seine estuary. France. *FEMS Microbiol. Ecol.* 55 230–238. 10.1111/j.1574.6941.2005.00021 16420631

[B52] Lenk S. Arnds J. Zerjatke K. Musat N. Amann R. Mußmann M. (2011). Novel groups of *Gammaproteobacteria* catalyse sulfur oxidation and carbon fixation in a coastal, intertidal sediment. *Environ. Microbiol.* 13 758–774. 10.1111/j.1462-2920.2010.02380.x 21134098

[B53] Li H. Jubelirer S. Garcia Costas A. M. Frigaard N.-U. Bryant D. A. (2009). Multiple antioxidant proteins protect *Chlorobaculum tepidum* against oxygen and reactive oxygen species. *Arch. Microbiol.* 191 853–867. 10.1007/s00203-009-0514-7 19784828

[B54] Liu Z. Frigaard N.-U. Vogl K. Iino T. Ohkuma M. Overmann J. (2012a). Complete genome of *Ignavibacterium album*, a metabolically versatile, flagellated, facultative anaerobe from the phylum *Chlorobi*. *Front. Microbiol.* 3:185. 10.3389/fmicb.2012.00185 22661972 PMC3362086

[B55] Liu Z. Klatt C. G. Ludwig M. Rusch D. B. Jensen S. I. Kühl M. (2012b). “*Candidatus* Thermochlorobacter aerophilum:” an aerobic chlorophotoheterotrophic member of the phylum Chlorobi defined by metagenomics and metatranscriptomics. *ISME J.* 6 1869–1882. 10.1038/ismej.2012.24 22456447 PMC3446795

[B56] Liu Z. Klatt C. G. Wood J. M. Rusch D. B. Ludwig M. Wittekindt N. (2011). Metatranscriptomic analyses of chlorophototrophs of a hot-spring microbial mat. *ISME J.* 5 1279–1290. 10.1038/ismej.2011.37 21697962 PMC3146272

[B57] Llorens-Marès T. Liu Z. Allen L. Z. Rusch D. B. Craig M. T. Dupont C. L. (2017). Speciation and ecological success in dimly lit waters: horizontal gene transfer in a green sulfur bacteria bloom unveiled by metagenomic assembly. *ISME J.* 11 201–211. 10.1038/ismej.2016.93 27392085 PMC5315485

[B58] Love M. I. Huber W. Anders S. (2014). Moderated estimation of fold change and dispersion for RNA-seq data with DESeq2. *Genome Biol.* 15:550. 10.1186/s13059-014-0550-8 25516281 PMC4302049

[B59] Loy A. Duller S. Baranyi C. Mußmann M. Ott J. Sharon I. (2009). Reverse dissimilatory sulfite reductase as phylogenetic marker for a subgroup of sulfur-oxidizing prokaryotes. *Environ. Microbiol.* 11 289–299. 10.1111/j.1462-2920.2008.01760.x 18826437 PMC2702494

[B60] Lübbe Y. J. Youn H. S. Timkovich R. Dahl C. (2006). Siro(haem)amide in *Allochromatium vinosum* and relevance of DsrL and DsrN, a homolog of cobyrinic acid a,c-diamide synthase, for sulphur oxidation. *FEMS Microbiol. Lett.* 261 194–202. 10.1111/j.1574-6968.2006.00343.x 16907720

[B61] Ludwig W. Strunk O. Westram R. Richter L. Meier H. Yadhukumar Buchner A. (2004). ARB: a software environment for sequence data. *Nucleic Acids Res.* 32 1363–1371. 10.1093/nar/gkh293 14985472 PMC390282

[B62] Magoc T. Wood D. Salzberg S. L. (2013). EDGE-pro: estimated degree of gene expression in prokaryotic genomes. *Evol. Bioinform. Online* 9 127–136. 10.4137/EBO.S11250 23531787 PMC3603529

[B63] Marchler-Bauer A. Derbyshire M. K. Gonzales N. R. Lu S. Chitsaz F. Geer L. Y. (2015). CDD: NCBI’s conserved domain database. *Nucleic Acids Res.* 43 D222–D226. 10.1093/nar/gku1221 25414356 PMC4383992

[B64] Markowitz V. M. Chen I. M. A. Chu K. Szeto E. Palaniappan K. Pillay M. (2014). IMG/M 4 version of the integrated metagenome comparative analysis system. *Nucleic Acids Res.* 42 D568–D573. 10.1093/nar/gkt919 24136997 PMC3964948

[B65] Martin M. (2011). Cutadapt removes adapter sequences from high-throughput sequencing reads. *EMBnet J.* 17 10–12. 10.14806/ej.17.1.200

[B66] Mavromatis K. Ivanova N. N. Chen I. M. Szeto E. Markowitz V. M. Kyrpides N. C. (2009). The DOE-JGI standard operating procedure for the annotations of microbial genomes. *Stand. Genomic. Sci.* 1 63–67. 10.4056/sigs.632 21304638 PMC3035208

[B67] McCleskey R. B. Ball J. W. Nordstrom D. K. Halloway J. M. Taylor H. E. (2004). *Water-Chemistry data for Selected Springs, Geysers and Streams in Yellowstone National Park, Wyoming, 2001-2002.* Reston, VA: United States Geological Survey 10.5066/F7M043FS

[B68] Meyer B. Kuevert J. (2007). Phylogeny of the alpha and beta subunits of the dissimilatory adenosine-5’ -phosphosulfate (APS) reductase from sulfate-reducing prokaryotes - Origin and evolution of the dissimilatory sulfate-reduction pathway. *Microbiology* 153 2026–2044. 10.1099/mic.0.2006/003152-0 17600048

[B69] Moreau J. W. Zierenberg R. A. Banfield J. F. (2010). Diversity of dissimilatory sulfite reductase genes (*dsrAB*) in a salt marsh impacted by long-term acid mine drainage. *Appl. Environ. Microbiol.* 76 4819–4828. 10.1128/AEM.03006-09 20472728 PMC2901737

[B70] Mori Y. Purdy K. J. Oakley B. B. Kondo R. (2010). Comprehensive detection of phototrophic sulfur bacteria using PCR primers that target reverse dissimilatory sulfite reductase gene. *Microbes Environ.* 25 190–196. 10.1264/jsme2.ME10109 21576872

[B71] Müller A. L. Kjeldsen K. U. Rattei T. Pester M. Loy A. (2015). Phylogenetic and environmental diversity of DsrAB-type dissimilatory (bi)sulfite reductases. *ISME J.* 9 1152–1165. 10.1038/ismej.2014.208 25343514 PMC4351914

[B72] Mussmann M. Richter M. Lombardot T. Meyerdierks A. Kuever J. Kube M. (2005). Clustered genes related to sulfate respiration in uncultured prokaryotes support the theory of their concomitant horizontal transfer. *J. Bacteriol.* 187 7126–7137. 10.1128/JB.187.20.7126 16199583 PMC1251608

[B73] Nakagawa T. Ishibashi J. I. Maruyama A. Yamanaka T. Morimoto Y. Kimura H. (2004). Analysis of dissimilatory sulfite reductase and 16S rRNA gene fragments from deep-sea hydrothermal sites of the Suiyo Seamount, Izu-Bonin Arc, Western Pacific. *Appl. Environ. Microbiol.* 70 393–403. 10.1128/AEM.70.1.393-403.2004 14711668 PMC321305

[B74] Nikolenko S. I. Korobeynikov A. I. Alekseyev M. A. (2013). BayesHammer: Bayesian clustering for error correction in single-cell sequencing. *BMC Genom.* 14:S7. 10.1186/1471-2164-14-S1-S7 23368723 PMC3549815

[B75] Nishihara A. Haruta S. E. McGlynn S. Thiel V. Matsuura K. (2018). Nitrogen fixation in thermophilic chemosynthetic microbial communities depending on hydrogen, sulfate, and carbon dioxide. *Microbes Environ.* 11 10–18. 10.1264/jsme2.ME17134 29367473 PMC5877335

[B76] Nunoura T. Hirayama H. Takami H. Oida H. Nishi S. Shimamura S. (2005). Genetic and functional properties of uncultivated thermophilic crenarchaeotes from a subsurface gold mine as revealed by analysis of genome fragments. *Environ. Microbiol.* 7 1967–1984. 10.1111/j.1462-2920.2005.00881.x 16309394

[B77] Oren A. Da Costa M. S. Garrity G. M. Rainey F. A. Rosselló-Móra R. Schink B. (2015). Proposal to include the rank of phylum in the international code of nomenclature of prokaryotes. *Int. J. Syst. Evol. Microbiol.* 65 4284–4287. 10.1099/ijsem.0.000664 26654112

[B78] Overbeek R. Olson R. Pusch G. D. Olsen G. J. Davis J. J. Disz T. (2014). The SEED and the Rapid Annotation of microbial genomes using Subsystems Technology (RAST). *Nucleic Acids Res.* 42 D206–D214. 10.1093/nar/gkt1226 24293654 PMC3965101

[B79] Parenteau M. N. Cady S. L. (2010). Microbial Biosignatures in iron-mineralized phototrophic mats at Chocolate Pots hot springs, Yellowstone National Park, United States. *Palaios* 25 97–111. 10.2110/palo.2008.p08-133r

[B80] Parks D. H. Imelfort M. Skennerton C. T. Hugenholtz P. Tyson G. W. (2015). CheckM: assessing the quality of microbial genomes recovered from. *Genome Res.* 25 1043–1055. 10.1101/gr.186072.114 25977477 PMC4484387

[B81] Pelikan C. Herbold C. W. Hausmann B. Müller A. L. Pester M. Loy A. (2016). Diversity analysis of sulfite- and sulfate-reducing microorganisms by multiplex *dsrA* and *dsrB* amplicon sequencing using new primers and mock community-optimized bioinformatics. *Environ. Microbiol.* 18 2994–3009. 10.1111/1462-2920.13139 26625892

[B82] Pester M. Bittner N. Deevong P. Wagner M. Loy A. (2010). A “rare biosphere” microorganism contributes to sulfate reduction in a peatland. *ISME J.* 4 1591–1602. 10.1038/ismej.2010.75 20535221 PMC4499578

[B83] Pester M. Knorr K. H. Friedrich M. W. Wagner M. Loy A. (2012). Sulfate-reducing microorganisms in wetlands - fameless actors in carbon cycling and climate change. *Front. Microbiol.* 3:72. 10.3389/fmicb.2012.00072 22403575 PMC3289269

[B84] Pires R. H. Lourenço A. I. Morais F. Teixeira M. Xavier A. V. Saraiva L. M. (2003). A novel membrane-bound respiratory complex from *Desulfovibrio desulfuricans* ATCC 27774. *Biochim. Biophys. Acta Bioenerg.* 1605 67–82. 10.1016/S0005-2728(03)00065-3 12907302

[B85] Podosokorskaya O. A. Kadnikov V. V. Gavrilov S. N. Mardanov A. V. Merkel A. Y. Karnachuk O. V. (2013). Characterization of *Melioribacter roseus* gen. nov., sp. nov., a novel facultatively anaerobic thermophilic cellulolytic bacterium from the class *Ignavibacteria*, and a proposal of a novel bacterial phylum *Ignavibacteriae*. *Environ. Microbiol.* 15 1759–1771. 10.1111/1462-2920.12067 23297868

[B86] Rabus R. Hansen T. A. Widdel F. (2013). “Dissimilatory sulfate- and sulfur-reducing prokatyotes,” in *The Prokaryotes: Prokaryotic Physiology and Biochemistry*, eds Rosenberg E. Delong E. F. Stackebrandt E. Lory S. Thompson F. (Berlin: Springer), 310–404. 10.1007/978-3-642-30141-4_70

[B87] Raina S. Missiakas D. Georgopoulos C. (1995). The *rpoE* gene encoding the sigma E (sigma 24) heat shock sigma factor of *Escherichia coli*. *EMBO J.* 14 1043–1055. 10.1002/j.1460-2075.1995.tb07085.x 7889935 PMC398177

[B88] Saldanha A. J. (2004). Java Treeview-extensible visualization of microarray data. *Bioinformatics* 20 3246–3248. 10.1093/bioinformatics/bth349 15180930

[B89] Santos A. A. Venceslau S. S. Grein F. Leavitt W. D. Dahl C. Johnston D. T. (2015). A protein trisulfide couples dissimilatory sulfate reduction to energy conservation. *Science* 350 1541–1545. 10.1126/science.aad3558 26680199

[B90] Sander J. Engels-Schwarzlose S. Dahl C. (2006). Importance of the DsrMKJOP complex for sulfur oxidation in *Allochromatium vinosum* and phylogenetic analysis of related complexes in other prokaryotes. *Arch. Microbiol.* 186 357–366. 10.1007/s00203-006-0156-y 16924482

[B91] Schoeffler M. Gaudin A.-L. Ramel F. Valette O. Denis Y. Hania W. B. (2018). Growth of an anaerobic sulfate-reducing bacterium sustained by oxygen respiratory energy conservation after O_2_-driven experimental evolution. *Environ. Microbiol.* 10.1111/1462-2920.14466 [Epub ahead of print]. 30394641

[B92] Schut G. J. Adams M. W. W. (2009). The iron-hydrogenase of *Thermotoga maritima* utilizes ferredoxin and NADH synergistically: a new perspective on anaerobic hydrogen production. *J. Bacteriol.* 191 4451–4457. 10.1128/JB.01582-08 19411328 PMC2698477

[B93] Seemann T. (2014). Prokka: rapid prokaryotic genome annotation. *Bioinformatics* 30 2068–2069. 10.1093/bioinformatics/btu153 24642063

[B94] Shen Y. Buick R. Canfield D. (2001). Isotopic evidence for microbial sulphate reduction in the early Archaean era. *Nature* 410 77–81. 10.1038/35065071 11242044

[B95] Smith S. A. Dunn C. W. (2008). Phyutility: a phyloinformatics tool for trees, alignments and molecular data. *Bioinformatics* 24 715–716. 10.1093/bioinformatics/btm619 18227120

[B96] Speth D. R. In’T Zandt M. H. Guerrero-Cruz S. Dutilh B. E. Jetten M. S. M. (2016). Genome-based microbial ecology of anammox granules in a full-scale wastewater treatment system. *Nat. Commun.* 7. 10.1038/ncomms11172 27029554 PMC4821891

[B97] Steunou A.-S. Bhaya D. Bateson M. M. Melendrez M. C. Ward D. M. Brecht E. (2006). *In situ* analysis of nitrogen fixation and metabolic switching in unicellular thermophilic cyanobacteria inhabiting hot spring microbial mats. *Proc. Natl. Acad. Sci. U.S.A.* 103 2398–2403. 10.1073/pnas.0507513103 16467157 PMC1413695

[B98] Takami H. Noguchi H. Takaki Y. Uchiyama I. Toyoda A. Nishi S. (2012). A deeply branching thermophilic bacterium with an ancient acetyl-CoA pathway dominates a subsurface ecosystem. *PLoS One* 7:e30559. 10.1371/journal.pone.0030559 22303444 PMC3267732

[B99] Tank M. Bryant D. A. (2015a). *Chloracidobacterium thermophilum* gen. nov., sp. nov.: an anoxygenic microaerophilic chlorophotoheterotrophic acidobacterium. *Int. J. Syst. Evol. Microbiol.* 65 1426–1430. 10.1099/ijs.0.000113 25667398

[B100] Tank M. Bryant D. A. (2015b). Nutrient requirements and growth physiology of the photoheterotrophic Acidobacterium, *Chloracidobacterium thermophilum*. *Front. Microbiol.* 6:226. 10.3389/fmicb.2015.00226 25870589 PMC4376005

[B101] Tank M. Thiel V. Ward D. M. Bryant D. A. (2017). “A panoply of phototrophs: an overview of the thermophilic chlorophototrophs of the microbial mats of alkaline siliceous hot springs in Yellowstone National Park, WY, USA,” in *Modern Topics in the Phototrophic Prokaryotes*, ed. Hallenbeck P. (Cham: Springer). 10.1007/978-3-319-46261-5

[B102] Thiel V. Hamilton T. L. Tomsho L. P. Burhans R. Gay S. E. Ramaley R. F. (2014). Draft genome sequence of the moderately thermophilic bacterium *Schleiferia thermophila* strain Yellowstone (*Bacteroidetes*). *GenomeA* 2:e00860–14. 10.1128/genomeA.00860-14 25169864 PMC4148732

[B103] Thiel V. Hügler M. Ward D. M. Bryant D. A. (2017). The dark side of the Mushroom Spring microbial mat: life in the shadow of chlorophototrophs. II. metabolic functions of abundant community members predicted from metagenomic analyses. *Front. Microbiol.* 8:943. 10.3389/fmicb.2017.00943 28634470 PMC5459899

[B104] Thiel V. Wood J. M. Olsen M. T. Tank M. Klatt C. G. Ward D. M. (2016). The dark side of the mushroom spring microbial mat: life in the shadow of chlorophototrophs. I. Microbial diversity based on 16S rRNA gene amplicons and metagenomic sequencing. *Front. Microbiol.* 7:919. 10.3389/fmicb.2016.00919 27379049 PMC4911352

[B105] Thompson J. D. Higgins D. G. Gibson T. J. (1994). CLUSTAL W: improving the sensitivity of progressive multiple sequence alignment through sequence weighting, position-specific gap penalties and weight matrix choice. *Nucleic Acids Res.* 22 4673–4680. 10.1038/35065071 7984417 PMC308517

[B106] Thorup C. Schramm A. Findlay A. J. Finster K. W. Schreiber L. (2017). Disguised as a sulfate reducer: growth of the deltaproteobacterium *Desulfurivibrio alkaliphilus* by sulfide oxidation with nitrate. *mBio* 8:e00671–17. 10.1128/mBio.00671-17 28720728 PMC5516251

[B107] Ultsch A. Moerchen F. (2005). *ESOM-Maps: Tools for Clustering, Visualization, and Classification with Emergent SOM.* Technical Report No. 46 Marburg: University of Marburg, Germany.

[B108] van der Meer M. T. J. Schouten S. Bateson M. M. Nübel U. Wieland A. Kühl M. (2005). Diel variations in carbon metabolism by green nonsulfur-like bacteria in alkaline siliceous hot spring microbial mats from Yellowstone National Park. *Appl. Environ. Microbiol.* 71 3978–3986. 10.1128/AEM.71.7.3978-3986.2005 16000812 PMC1168979

[B109] Wacey D. Kilburn M. R. Saunders M. Cliff J. Brasier M. D. (2011). Microfossils of sulphur-metabolizing cells in 3.4-billion-year-old rocks of Western Australia. *Nat. Geosci.* 4 698–702. 10.1038/ngeo1238

[B110] Wagner M. Roger A. J. Flax J. L. Brusseau G. A. Stahl D. A. (1998). Phylogeny of dissimilatory sulfite reductases supports an early origin of sulfate respiration. *J. Bacteriol.* 180 2975–2982. 9603890 10.1128/jb.180.11.2975-2982.1998 PMC107267

[B111] Wang Z. Wu M. (2013). A phylum-level bacterial phylogenetic marker database. *Mol. Biol. Evol.* 30 1258–1262. 10.1093/molbev/mst059 23519313

[B112] Ward D. M. Bateson M. M. Ferris M. J. Kühl M. Wieland A. Koeppel A. (2006). Cyanobacterial ecotypes in the microbial mat community of Mushroom Spring (Yellowstone National Park, Wyoming) as species-like units linking microbial community composition, structure and function. *Philos. Trans. R. Soc. Lond. Ser. B Biol. Sci.* 361 1997–2008. 10.1098/rstb.2006.1919 17028085 PMC1764927

[B113] Ward D. M. Cohan F. M. Bhaya D. Heidelberg J. F. Kühl M. Grossman A. (2008). Genomics, environmental genomics and the issue of microbial species. *Heredity* 100 207–219. 10.1038/sj.hdy.6801011 17551524

[B114] Ward D. M. Ferris M. J. Nold S. C. Bateson M. M. (1998). A natural view of microbial biodiversity within hot spring cyanobacterial mat communities. *Microbiol. Mol. Biol. Rev.* 62 1353–1370. 9841675 10.1128/mmbr.62.4.1353-1370.1998 PMC98949

[B115] Weissgerber T. Sylvester M. Kröninger L. Dahl C. (2014). A comparative quantitative proteomic study identifies new proteins relevant for sulfur oxidation in the purple sulfur bacterium *Allochromatium vinosum*. *Appl. Environ. Microbiol.* 80 2279–2292. 10.1128/AEM.04182-13 24487535 PMC3993150

[B116] Wu M. Scott A. J. (2012). Phylogenomic analysis of bacterial and archaeal sequences with AMPHORA2. *Bioinformatics* 28 1033–1034. 10.1093/bioinformatics/bts079 22332237

[B117] Zang S. S. Jiang H. B. Song W. Y. Chen M. Qiu B. S. (2017). Characterization of the sulfur-formation (*suf*) genes in *Synechocystis* sp. PCC 6803 under photoautotrophic and heterotrophic growth conditions. *Planta* 246 927–938. 10.1007/s00425-017-2738-0 28710587

[B118] Zecchin S. Mueller R. C. Seifert J. Stingl U. Anantharaman K. von Bergen M. (2018). Rice paddy *Nitrospirae* carry and express genes related to sulfate respiration: proposal of the new genus “*Candidatus* Sulfobium.” *Appl. Environ. Microbiol.* 84 1–15. 10.1128/AEM.02224-17 29247059 PMC5812927

[B119] Zverlov V. Klein M. Lücker S. Friedrich M. W. Kellermann J. Stahl D. A. (2005). Lateral gene transfer of dissimilatory (bi) sulfite reductase revisited. *J. Bacteriol.* 187 2203–2208. 10.1128/JB.187.6.2203-2208.2005 15743970 PMC1064038

