# Supplementary figures and images for: “Candidatus Thermonerobacter thiotrophicus,” A Non-phototrophic Member of the Bacteroidetes/Chlorobi With Dissimilatory Sulfur Metabolism in Hot Spring Mat Communities
Source: Front Microbiol. 2019 Jan 9;9:3159. doi: 10.3389/fmicb.2018.03159 (PMC6338057; doi:10.3389/fmicb.2018.03159)

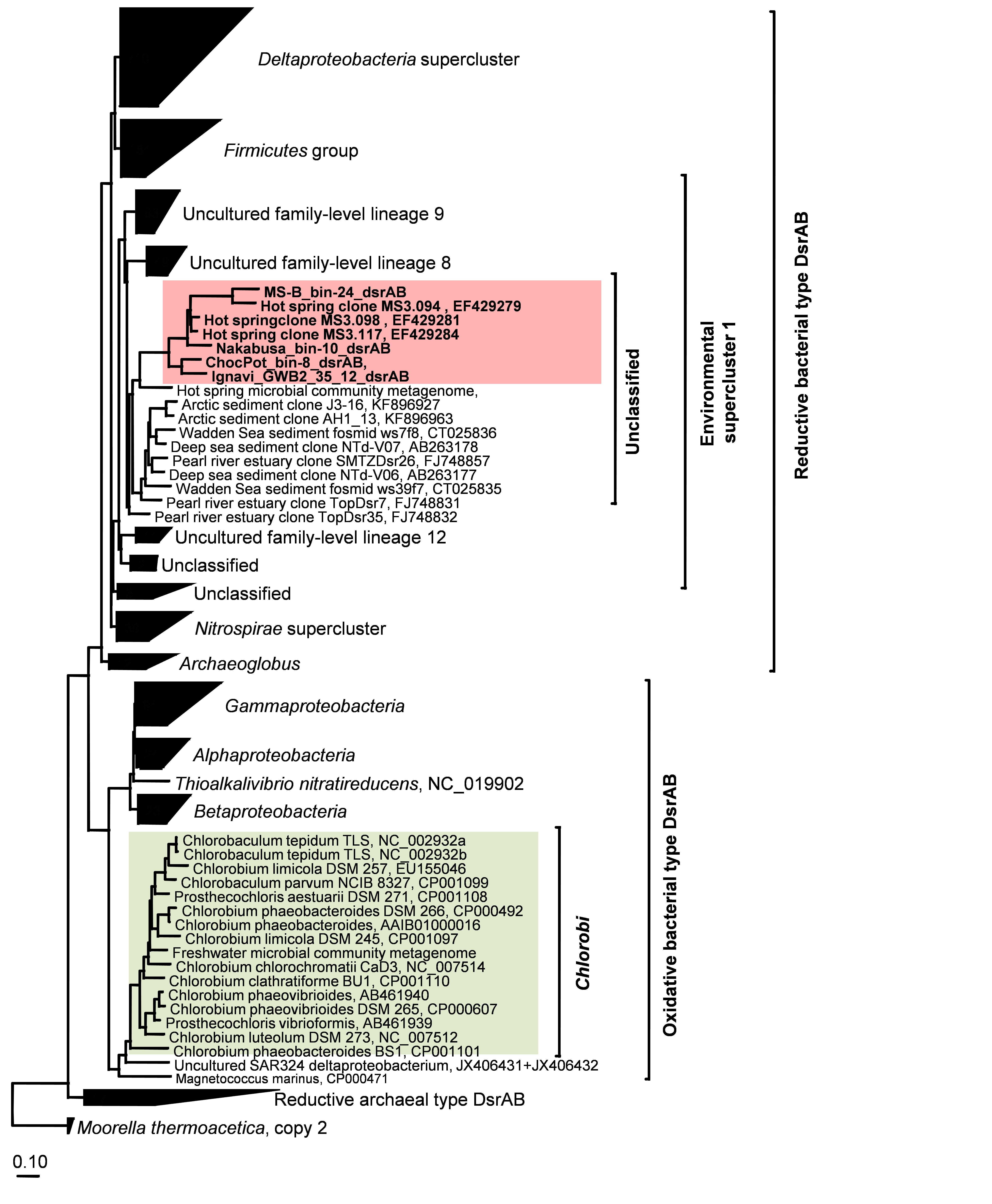

Supplement: Figure S1 — Phylogenetic tree based on DsrAB amino acid sequences displaying the phylogenetic affiliation of the putative thermophilic SRM Bacteroidetes–Chlorobi group member (red box within the Environmental Supercluster in the reductive-type DsrAB clade). Sequences of GSB, SOM members of the Chlorobi cluster with the rDSR (sulfur oxidizing) DsrAB clade. The tree was calculated from with the publicly available DsrAB ARB database (Müller et al., 2015; http://www.microbial-ecology.net/download). New sequences were added using the parsimony method without changing the tree topology. [file Image_1.TIF]

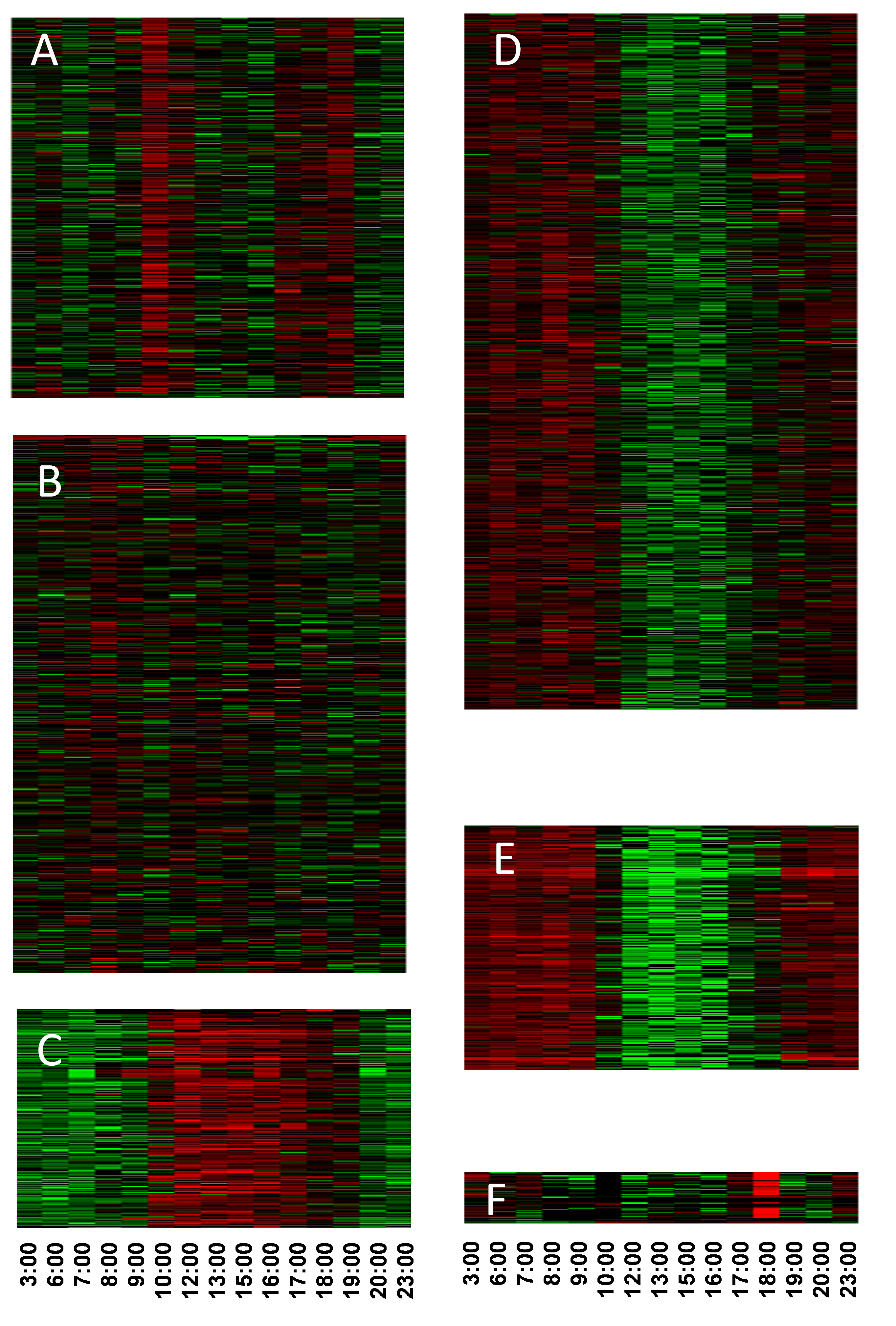

Supplement: Figure S2 — Heatmaps showing expression patterns of “Ca. T. thiotrophicus” genes throughout a diel cycle in the upper green layer of MS (sampling times shown at the bottom). Red = high relative transcript abundance; green = low relative transcript abundance; black = no transcripts detected. Higher color intensity reflects relatively higher transcript levels. Genes are clustered according to their major patterns of expression as described in the text. Red (A) Genes (390) with expression peaks at 09:00 and 18:00. (B) Genes (546) with variable expression throughout the day. (C) Genes (222) with strong daytime expression. (D) Genes (706) with weak nighttime expression. (E) Genes (248) with strong nighttime expression. (F) Genes (52) with maximal transcript levels at 18:00. [file Image_2.TIF]

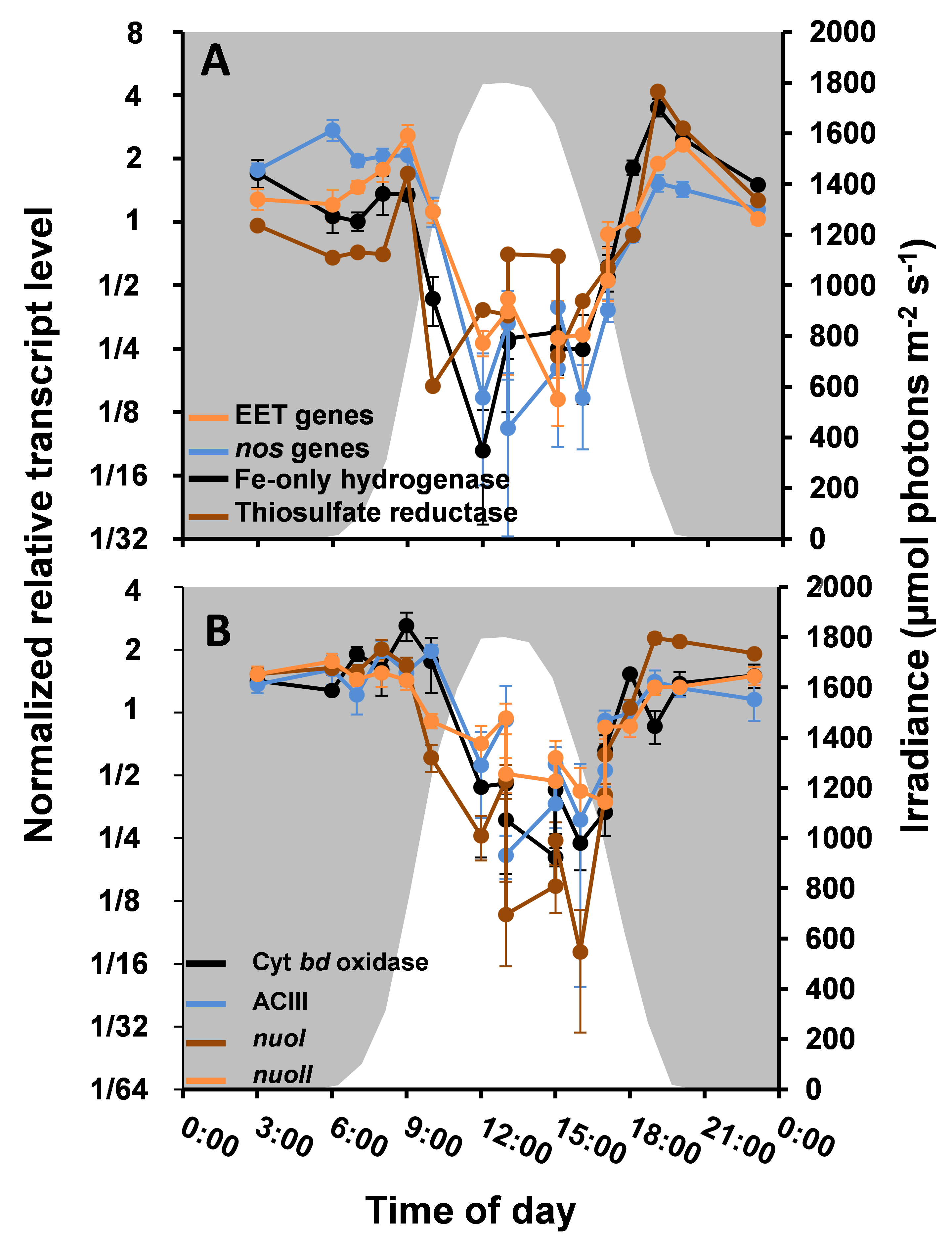

Supplement: Figure S3 — Relative transcript levels (y-axis) across a diel cycle (x-axis) for in the upper green layer of MS of genes associated with anaerobic respiration (A) and other electron transfer processes (B). White areas indicate light periods during the day and irradiance intensities in μmol photons m-2 s-1 (400–700 nm, secondary y-axis), while gray areas indicate dark periods during the night. [file Image_3.TIF]

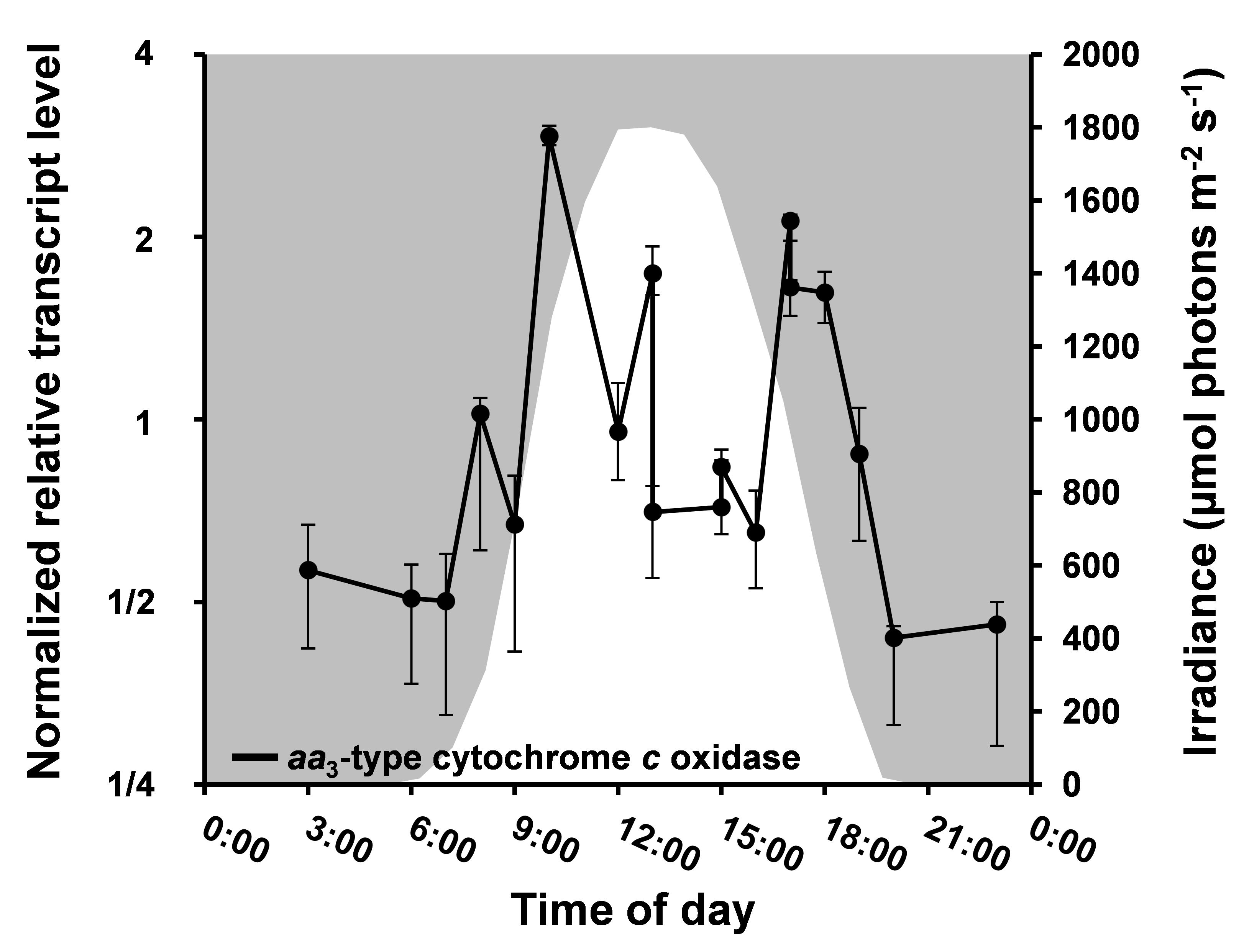

Supplement: Figure S4 — Relative transcript levels (x-axis) of aa3-type cytochrome c oxidase across a diel cycle (x-axis) in the upper green mat layer of MS. White areas indicate light periods during the day and irradiance intensities in μmol photons m-2 s-1 (400–700 nm, secondary y-axis), while gray areas indicate dark periods during the night. [file Image_4.TIF]

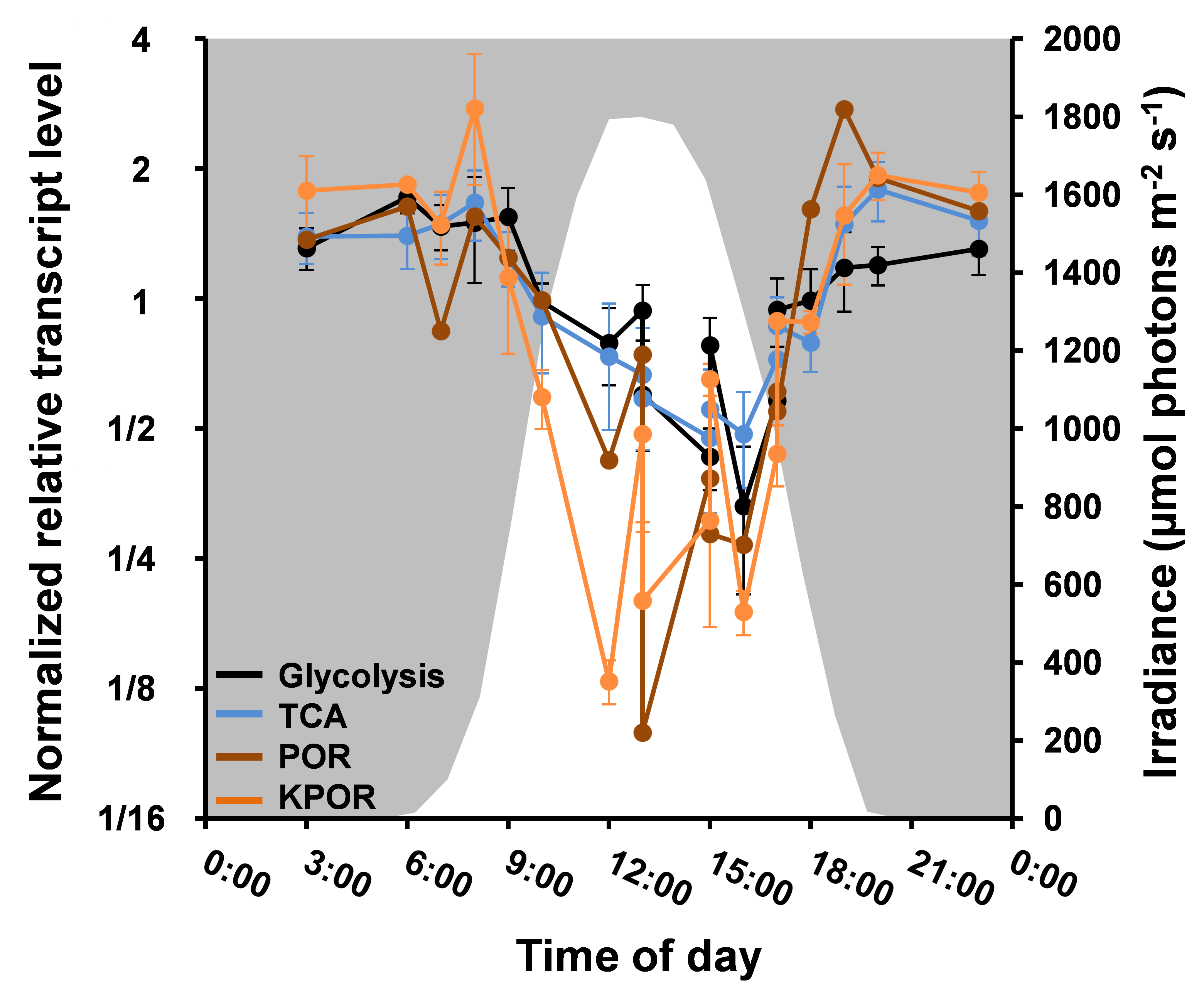

Supplement: Figure S5 — Relative transcription levels (y-axis) of genes involved in glycolysis, the TCA cycle, and other central carbon metabolism pathways across a diel cycle (x-axis) in MS MAG (MS-B_bin-24), in the upper green mat layer of MS. White areas indicate light periods during the day and irradiance intensities in μmol photons m-2 s-1 (400–700 nm, secondary y-axis), while gray areas indicate dark periods during the night. [file Image_5.TIF]

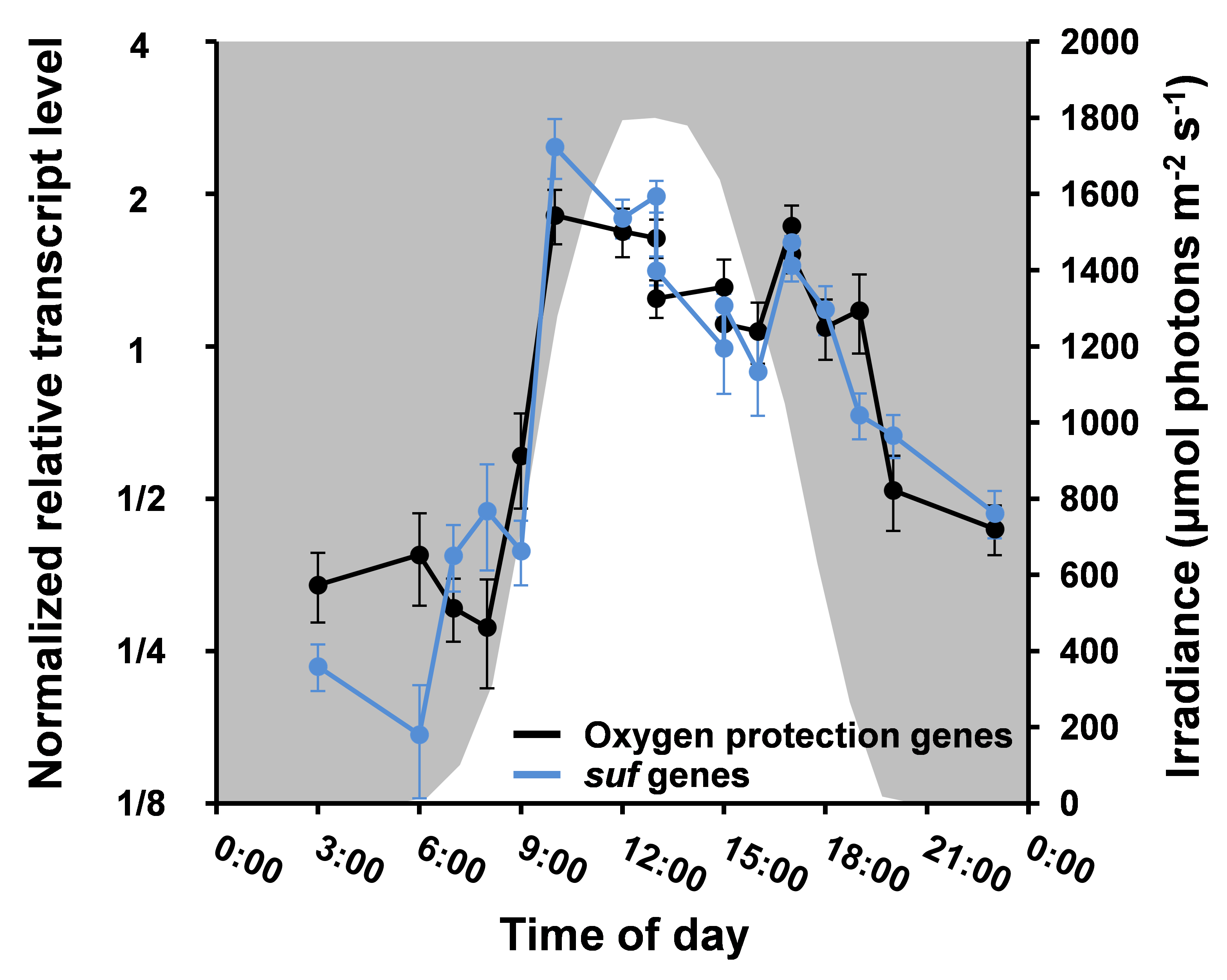

Supplement: Figure S6 — Relative transcript levels (y-axis) of genes associated with oxygen stress/oxic conditions during a diel cycle (y-axis) in the upper green mat layer of MS. White areas indicate light periods during the day and irradiance intensities in μmol photons m-2 s-1 (400–700 nm, secondary y-axis), while gray areas indicate dark periods during the night. [file Image_6.TIF]

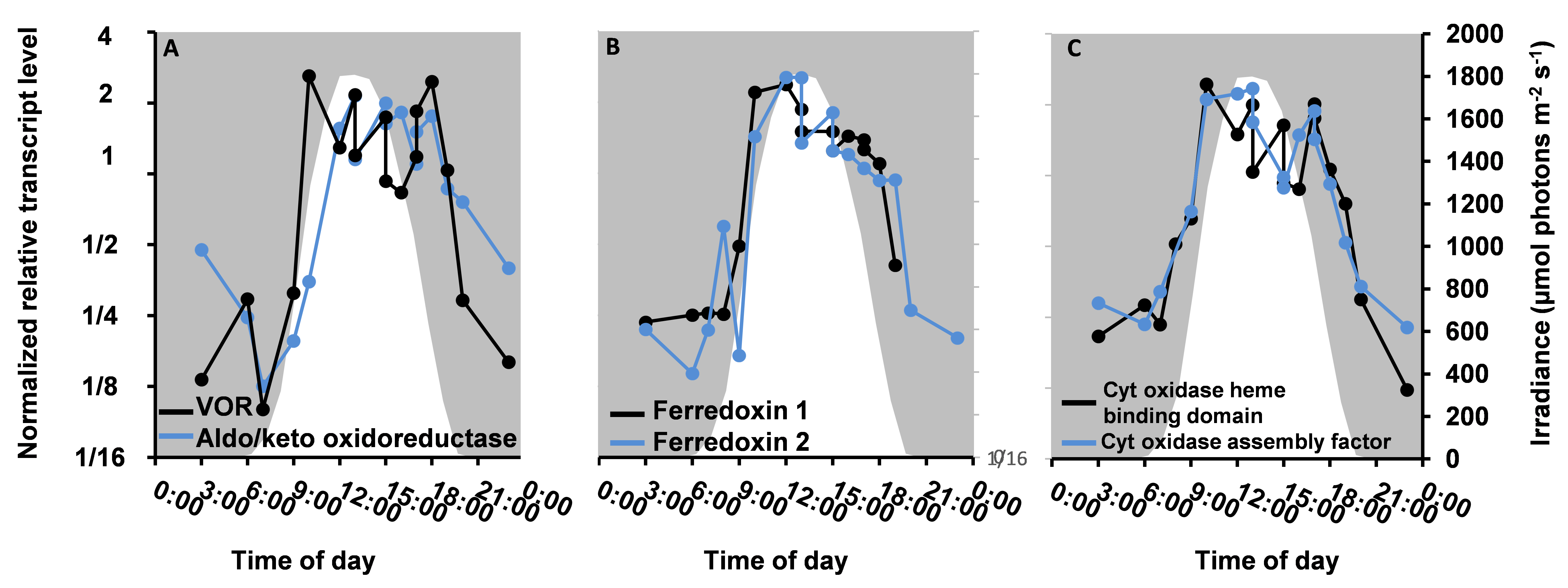

Supplement: Figure S7 — Relative transcript levels (y-axis) of selected day genes across a diel cycle (x-axis) in the upper green mat layer of MS. (A) Putative 2-oxovalerate:ferredoxin oxidoreductase (VOR) and aldo/keto oxidoreductase; (B) ferredoxins; (C) cytochrome biosynthesis genes. White areas indicate light periods during the day and irradiance intensities in μmol photons m-2 s-1 (400–700 nm, secondary y-axis), while gray areas indicate dark periods during the night. [file Image_7.TIF]
